# Highly efficient CRISPR-Cas9 base editing in *Bifidobacterium* with bypass of restriction modification systems

**DOI:** 10.1128/aem.01985-24

**Published:** 2025-03-10

**Authors:** Hung-Chun Lin, Wan-Chi Hsiao, Ya-Chen Hsu, Meng-Chieh Lin, Cheng-Chih Hsu, Mingzi M. Zhang

**Affiliations:** 1 Department of Chemistry, National Taiwan University 202948 , Taipei, Taiwan; 2 Institute of Biotechnology, National Tsing Hua University 594261 , Hsinchu, Taiwan; 3 Institute of Molecular and Genomic Medicine, National Health Research Institutes 603246 , Miaoli, Taiwan; 4 Leeuwenhoek Laboratories Co. Ltd, Taipei, Taiwan; University of Illinois Urbana-Champaign, Urbana, Illinois, USA

**Keywords:** *Bifidobacterium*, strain engineering, CRISPR-Cas, restriction modification systems, DNA methylation, metabolic perturbation

## Abstract

Intestinal microbiota members of the *Bifidobacterium* genus are increasingly explored as probiotics and therapeutics. However, the paucity of genetic tools and the widespread restriction modification (RM) systems in *Bifidobacterium* limit our ability to genetically manipulate these bacteria. Here we established a CRISPR-Cas9 cytosine base editor system (cBEST) for portable genome editing in bifidobacteria. Harboring different promoters characterized in this study, these cBEST plasmids showed a range of editing efficiencies in different strains and genomic contexts, highlighting the importance of fine-tuning base editor and sgRNA expression. Additionally, we showed that disruption or bypass of RM systems dramatically improved editing efficiencies in otherwise hard-to-edit genomic loci and *Bifidobacterium* strains. Notably, we demonstrated the use of RM-disrupted *Bifidobacterium longum* strains for simultaneous assembly, amplification, and methylation of the all-in-one editing plasmids, greatly streamlining the workflow for high-efficiency base editing. Last but not least, we showed the portability of cBESTs using the same editing construct to disrupt a conserved metabolic gene in multiple *Bifidobacterium* species. Looking ahead, the ability to efficiently edit and engineer bifidobacterial genomes will give rise to new opportunities for research and applications toward improving human health.

**IMPORTANCE:**

The ability to genetically manipulate specific genes and biological pathways in *Bifidobacterium* is essential to unlocking their probiotic and therapeutic potential in human health applications. The DNA double-strand break-free CRISPR-Cas9 cytosine base editor system established in this work allows portable and efficient base editing in *Bifidobacterium* spp. We further showed that bypass of restriction modification systems significantly improved base editing efficiency, especially for hard-to-edit genomic loci and strains. This expanded *Bifidobacterium* genome editing toolbox should facilitate mechanistic investigations into the roles of *Bifidobacterium* in host physiology and disease.

## INTRODUCTION

Advances in multi-omics analyses and bioinformatics have shed light on the crucial roles of human gut commensals in shaping human physiology and diseases (1, 2). Among the gut microbiota, *Bifidobacterium* stands out as a genus of interest commonly used as probiotics with its multitude of beneficial effects on human health, including regulation of immune response, enhancement of cancer immunotherapy, maintenance of gut barrier function, metabolic homeostasis, and shaping of gut microbiota composition at different life stages (3–7). Yet much of these studies are driven by the association of specific *Bifidobacterium* strains with particular host phenotypes, and causal relationships are usually established through inoculation of target strains or the use of synthetic microbial communities (8, 9).

The ability to genetically manipulate specific genes and biological pathways in target bacterial strains is critical to understanding the molecular mechanisms underpinning *Bifidobacteria*–host interactions. Despite the increasing number of publicly available bifidobacterial genome sequences, the paucity of genetic tools as well as widespread restriction modification (RM) systems in *Bifidobacterium* greatly limit our ability to genetically manipulate these bacteria (10, 11). Recently, endogenous type I CRISPR-Cas systems and homology-directed repair were harnessed for genome editing of *Bifidobacterium animalis* subsp. *lactis* and *Bifidobacterium breve* strains (12, 13), but this approach may not be feasible for strains without characterized CRISPR-Cas systems. Alternatively, exogenous type II CRISPR-Cas systems offer predictable and potentially portable genome editing but require the transformation of much larger editing plasmids. For example, the widely used CRISPR-Cas9 system from *Streptococcus pyogenes* (spCas9) was successfully used for genome editing in *B. animalis* AR668 (14). Nonetheless, these studies mainly focus on homology-directed repair of CRISPR-Cas generated DNA double-strand breaks (DSBs) in bifidobacterial genomes. DSB-dependent genome editing methods involving CRISPR-Cas nucleases generally allow more varied and complex edits such as indels, insertions, and large deletions, but they tend to be more error-prone with a higher risk of genomic instability and cytotoxicity resulting from unwanted genome modifications compared to DSB-independent editing techniques (15). While base editing technologies offer therapeutic opportunities to precisely correct pathogenic mutations in humans with multiple candidates entering clinical trials (16, 17), the use of base editors to generate targeted point mutations without the need for DSB generation or donor template DNA remains underexplored in *Bifidobacteria*.

In this study, we developed CRISPR-spCas9 cytosine base editor systems (cBESTs) with different promoter combinations for efficient DSB-free genome editing in multiple *Bifidobacteria* spp. and strains. We showed that disruption or bypass of native RM systems dramatically improved both transformation and genome editing efficiencies. We further demonstrated the use of the RM-disrupted strains as host strains for simultaneous amplification and methylation of *in vitro* assembled editing constructs, circumventing *Escherichia coli* as a cloning host and streamlining the cloning process while maintaining high editing efficiencies (60%–100%). Last but not least, we showcased the portability and utility of the base editor systems by using the same editing construct to knock out a conserved gene and achieve targeted metabolic perturbation in different *Bifidobacterium* strains.

## RESULTS

### Promoter characterization in *Bifidobacterium*

Precise gene expression is crucial for effective microbial bioengineering. However, there is only a limited number of characterized native *Bifidobacterium* promoters that can be used for heterologous gene expression (10, 11). Access to universal promoters of varied strengths, in particularly characterized minimally sized synthetic promoters will be key to building a genome editing toolbox for *Bifidobacteri*a.

**Fig 1:**
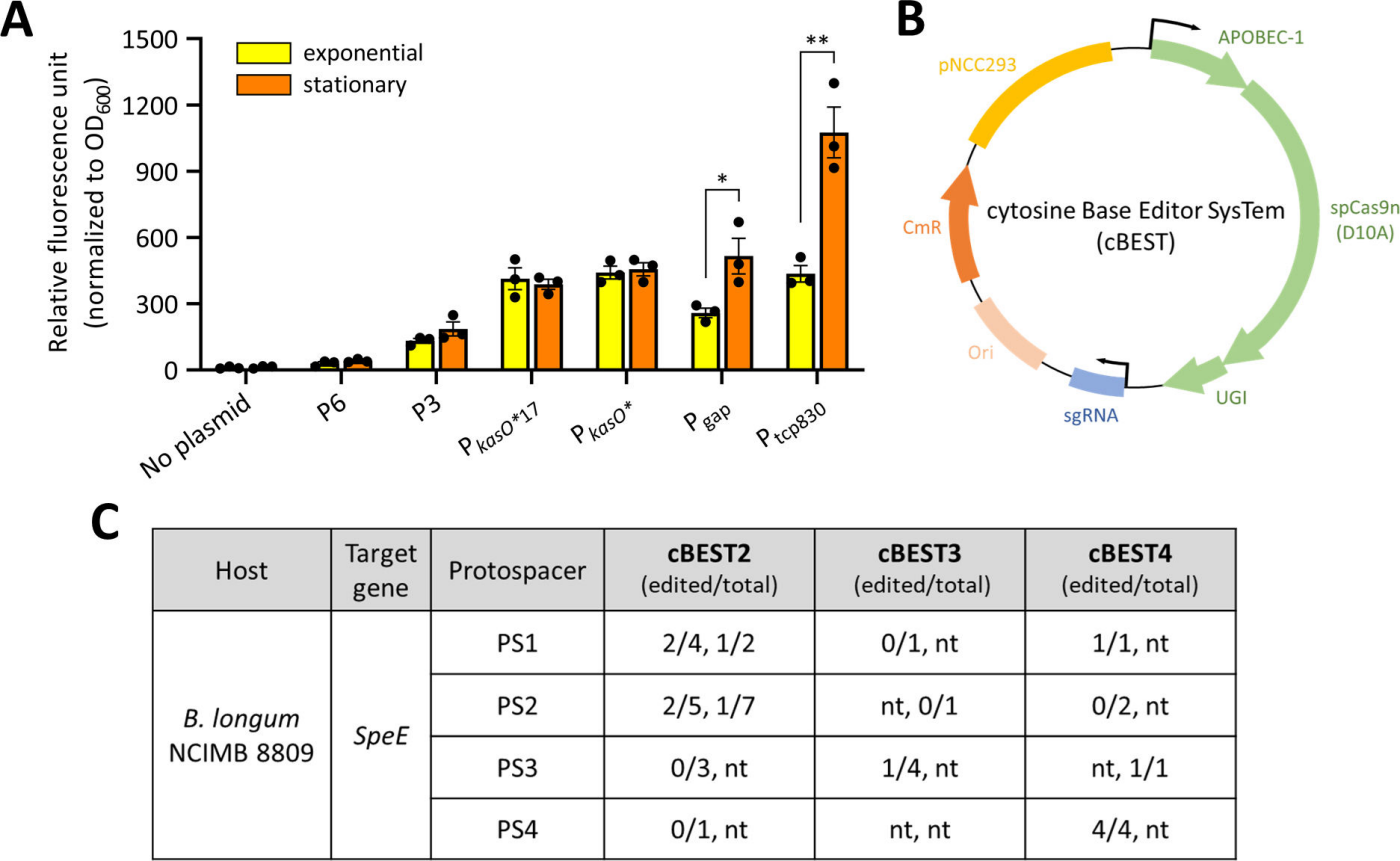
Construction and validation of three base editing constructs for *B. longum* NCIMB 8809. (**A**) Relative expression levels of various promoters in exponential and stationary phase cultures (*n* = 3; error bars, SEM). *P < 0.05, **P < 0.01. (**B**) Map of the cBEST plasmid containing both sgRNA and cytosine base editor. (**C**) Editing efficiencies of cBEST2, cBEST3, and cBEST4 targeting the *SpeE* gene with indicated promoters driving sgRNA and cytosine base editor expression, along with four protospacers (PS) in each row. Two independent experiments were performed for each construct. Editing efficiencies were expressed as (number of edited colonies / number of sequenced colonies) with data from independent transformations separated by commas. nt, no transformant obtained.

To identify promoters for the construction of genome editing systems, we first characterized the relative strengths of selected native and synthetic promoters in *Bifidobacterium longum* NCIMB 8809 (Fig. 1A). All constructs were derived from the pMGC-mCherry plasmid (Fig. S2A), which uses the *gap* gene promoter (P_gap_) from *Bifidobacterium bifidum* S17 to drive mCherry expression (18). Phylogenetically distant constitutive promoters from *Streptomyces* such as P3 and P6 (19, 20), which have been successfully used for heterologous gene expression in rare actinomycetes (21, 22), yielded detectable mCherry expression in *B. longum* NCIMB 8809, albeit at significantly lower levels compared to P_gap_. Among the synthetic promoters examined, the synthetic tetracycline-inducible tcp830 promoter (P_tcp830_) demonstrated the highest expression level (23). The synthetic kasO* promoter (P_kasO__*_) (24) and its variant, which harbor a shorter 17 bp spacer sequence between the −10 and −35 regions (P_kasO*__17_) (Fig. S2B) (25), displayed moderate expression levels similar to P_gap_. With the exception of P_gap_ and P_tcp830_, the other promoters tested yielded consistent mCherry expression during exponential and stationary growth .

To examine the portability of some of these promoters, we transformed these mCherry reporter plasmids into *Bifidobacterium adolescentis* DSM 20083. While overall mCherry fluorescence was higher in *B. adolescentis* DSM 20083 (Fig. S2C), the general trend of weak and strong promoters was similar to that observed in *B. longum* NCIMB 8809. Taken together, we identified promoters of varied strengths, including minimally sized synthetic promoters, which will be useful for controlling the expression of heterologous genes in *Bifidobacteria*.

### cBEST function in *B. longum* NCIMB 8809

Using the promoters characterized above, we constructed three sets of base editors to be tested in *B. longum* NCIMB 8809. Strong and moderate promoters (P_tcp830_ and P_kasO__*17tss_) were chosen to drive sgRNA expression. Considering the effect of Cas9 expression levels on cellular toxicity and genome editing efficiency in other bacteria (26, 27), moderate and relatively weak promoters (P_kasO__*_ and P3) were selected for expression of the base editor [(spCas9n (D10A) fused to APOBEC1 and UGI developed by Tong et al. (28)] (Fig. 1B). The three resulting cBEST plasmids (Table S6), named after their *Streptomyces* counterparts (28), are compatible with the one-pot Golden Gate assembly of protospacer sequences.

To test the functionality of the cBEST plasmids for genome editing in *Bifidobacterium*, we sought to disrupt the *SpeE* gene, which was predicted to be involved in the biosynthesis of 5′-methylthioadenosine (MTA) and possible protection against obesity (8). Four protospacers targeting regions within the *SpeE* gene were selected, yielding a total of 12 constructs (Fig. 1C), which were transformed into *B. longum* NCIMB 8809, and gene editing efficiencies were determined as the proportion of desired base-edited transformants to the total sequenced transformants (Fig. 1C). Given the small number of transformants obtained with each transformation, all transformants obtained were analyzed by Sanger sequencing, which confirmed C-to-T edits within the expected editing window of 11–17 nucleotides distal to the PAM sequence in plasmid-cured transformants (Fig. S3). Among the cBEST constructs, cBEST4 exhibited higher overall editing efficiencies with a total of six out of eight (75%) edited transformants compared to cBEST2 (6 of 22 = 27.2%) and cBEST3 (1 of 6 = 16.7%) over eight independent transformations involving four different protospacer sequences (Fig. 1C). These results suggested the relevance of fine-tuning base editor and sgRNA expression to achieve efficient genome editing.

Next, we conducted liquid chromatography–tandem mass spectrometry (LC-MS/MS) metabolic analysis to evaluate the impact of the *SpeE*(Q198*) strain on MTA production (Fig. S4). Methionine, S-adenosyl-L-methionine (SAM), and MTA were successfully detected, with confident tandem mass spectrometry fragmentation (Fig. S4E and F). Surprisingly, no regulation of MTA levels was observed in the *SpeE*(Q198*) strain (Fig. S4D). The intermediate product, dcSAM, the substrate for *SpeE* to produce MTA, was not detected. While alternative, unreported enzymes may be involved in MTA production, we cannot rule out the possibility of residual SpeE expression or function in the *SpeE*(Q198*) strain due to biological plasticity such as translational reinitiation or ribosome skipping (29, 30), which needs to be confirmed through additional experiments.

### Enhanced transformation efficiencies in RM-disrupted strains

Given that the cBEST plasmids yielded very few transformants (<10 CFU/µg plasmids) compared to reporter plasmids like pMGC-mCherry (Fig. S1B), we postulated that in addition to Cas-associated toxicity (31), RM systems and their roles in restricting foreign DNA may be the major bottleneck for editing bifidobacterial genomes. RM systems differentiate foreign DNA from endogenous genomic DNA through two main enzymatic components: modification methyltransferase (MTase) and restriction endonuclease (REase). The MTase protects DNA by adding a methyl group to cytosine or adenine, whereas the REase cleaves the specific sequences lacking methylation. RM systems are categorized based on the protein composition and recognition sequences among other factors (32, 33), and single molecule real-time sequencing has revealed diverse methylomes and RM systems across bacterial strains, including *Bifidobacterium* spp. (34, 35).

To elucidate the contribution of RM systems toward transformation and gene editing efficiencies in *B. longum* NCIMB 8809, we disrupted its native RM systems. Based on pan-genome analysis of *Bifidobacterium* RM systems and their predicted recognition motifs, we knocked out three REase genes (*HsdR*, *EcoRII_0606*, and *EcoRII_0983*) in *B. longum* NCIMB 8809 individually and in combination using cBEST plasmids (Fig. S5A through C). No growth differences were observed for the five REase mutant strains, including *HsdR*(W29*), *0606*(Q64*), *0983*(W138*), and REase double mutant *0606*(Q64*)*0983*(W138*) as well as an REase triple mutant *0606*(Q64*)*0983*(W138*)*HsdR*(W29*) (Fig. S5D). Additionally, based on their chloramphenicol sensitivities and colony PCR (Fig. S5 E and F), all cBEST-transformed strains could be cured of the cBEST plasmids after several rounds of propagation in selection-free medium, which was critical for sequential base editing to generate the REase double and triple mutants.

**Fig 2:**
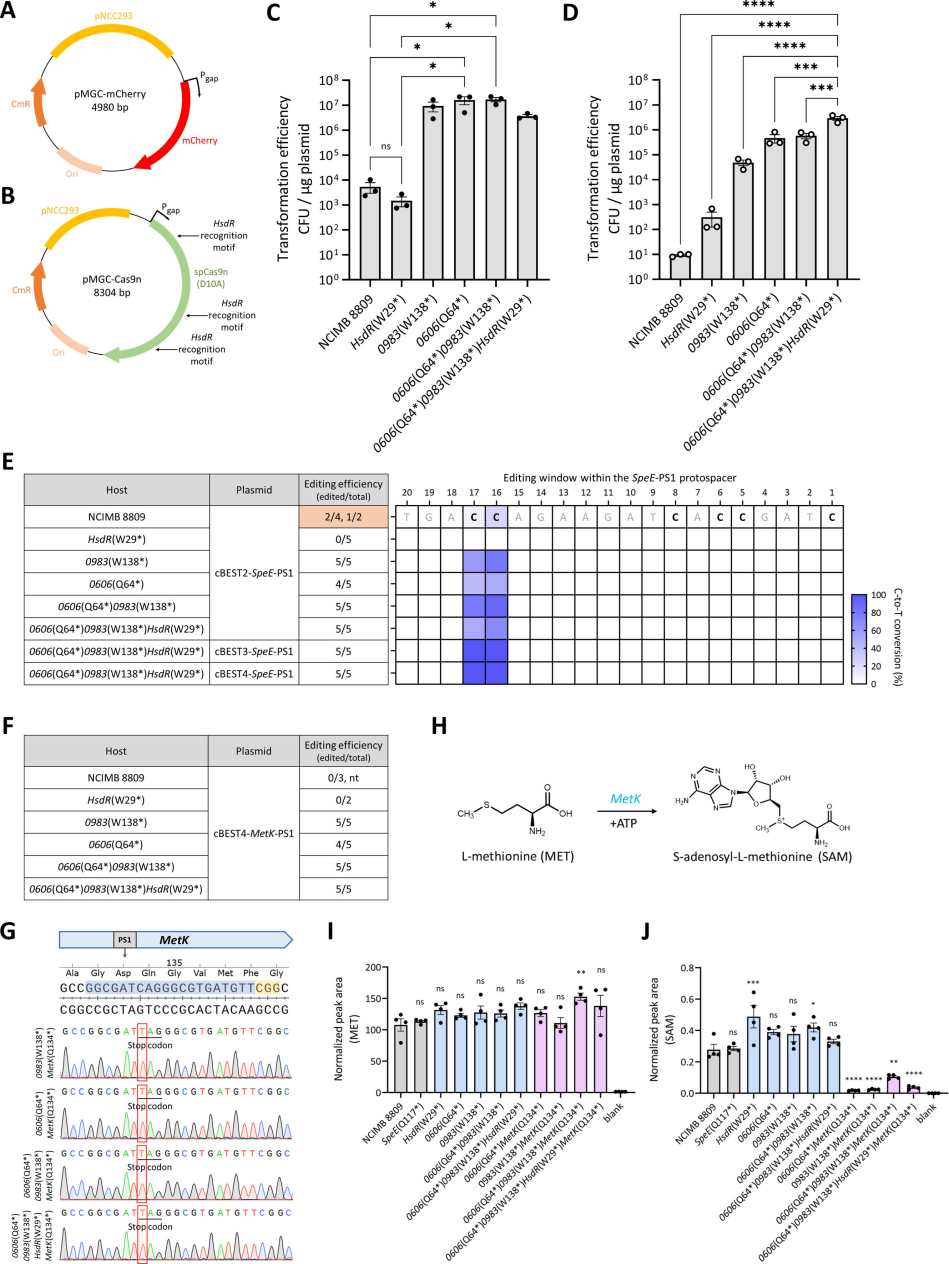
Assessment of all REase mutant strains derived from *B. longum* NCIMB 8809 through transformation and gene editing efficiencies. Maps of (**A**) pMGC-mCherry and (**B**) pMGC-Cas9n plasmids. Comparison of transformation efficiencies across indicated REase mutant strains using (**C**) pMGC-mCherry and (**D**) pMGC-Cas9n plasmids. (*n* = 3, biological triplicates; error bars, SEM). *P < 0.05, ***P < 0.001, ****P < 0.0001. (**E and F**) Editing efficiencies were expressed as (number of edited colonies / number of sequenced colonies) with data from independent transformations separated by commas, and editing windows were indicated for each tested condition. Five colonies were randomly picked for analysis for experiments with more than five transformants. Data shaded orange were first presented in Fig. 1C and reproduced here to facilitate comparison between the different strains. (**G**) Successful *MetK*(Q134*) mutant strains using the cBEST4-*MetK*-PS1 plasmid. Sequencing results confirmed precise introduction of the stop codon due to successful C-to-T edits (red frames). (**H**) *MetK* generates SAM from MET and ATP. (**I and J**) Normalized peak areas for each indicated metabolite in the indicated engineered *B. longum* NCIMB 8809 strains. (*n* = 4, biological replicates; error bars, SEM). *P < 0.05, **P < 0.01, ***P < 0.001, ****P < 0.0001. ns, not significant.

As expected, if the three predicted RM systems were active and played a major role in restricting foreign DNA in *B. longum* NCIMB 8809, we observed significantly improved transformation efficiencies ranging from one to five orders of magnitude with the REase knockout strains, depending on the plasmid used (Fig. 2A through D). Transformation efficiency of pMGC-Cas9n, which harbored *HsdR* recognition sequences within the Cas9n gene (Fig. 2B), was improved ~30-fold in the *HsdR*(W29*) strain (Fig. 2D). Underscoring the specificity of the RM systems, transformation efficiency of pMGC-mCherry, which did not contain *HsdR* recognition sequences, remained unchanged in the *HsdR*(W29*) strain compared to the parent wild-type strain (Fig. 2C). Notably, the *0606*(Q64*)*0983*(W138*)*HsdR*(W29*) strain showed the highest improvement in transformation efficiency of ~300,000-fold for pMGC-Cas9n compared to wild type (Fig. 2D), reaching efficiencies of 10^6^–10^7^ CFU/µg. These results revealed the major roles of the three RM systems in restricting the influx of foreign DNA, especially large plasmids such as pMGC-Cas9n, in *B. longum* NCIMB 8809.

### Enhanced gene editing efficiencies in RM-disrupted strains

To elucidate the contribution of RM systems toward genome editing efficiencies, we evaluated gene editing efficiencies of the cBEST2-*SpeE*-PS1 plasmid in the different *B. longum* NCIMB 8809 REase mutant strains (Fig. 2E; Fig. S6A through E). In general, both transformation and genome editing efficiencies were dramatically enhanced in the RM-disrupted strains. In addition to a larger number of transformants observed for the *0606*(Q64*)*0983*(W138*)*HsdR*(W29*) strain, all (15 of 15) randomly picked transformants possessed C-to-T edits confined to the expected editing window for three different cBEST constructs (Fig. 2E; Fig. S6 E through G). Further supporting the enhanced editing efficiencies in RM-disrupted strains, up to 100% of five randomly picked RM-disrupted transformants yielded the desired *MetK*(Q134*) mutation, which was challenging to attain with the wild-type *B. longum* NCIMB 8809 strain (Fig. 2F and G; Fig. S7). Furthermore, consistent with the functional disruption of MetK function, *MetK*(Q134*) cells showed significantly reduced levels of SAM despite similar levels of L-methionine (Fig. 2H through J).

**Fig 3:**
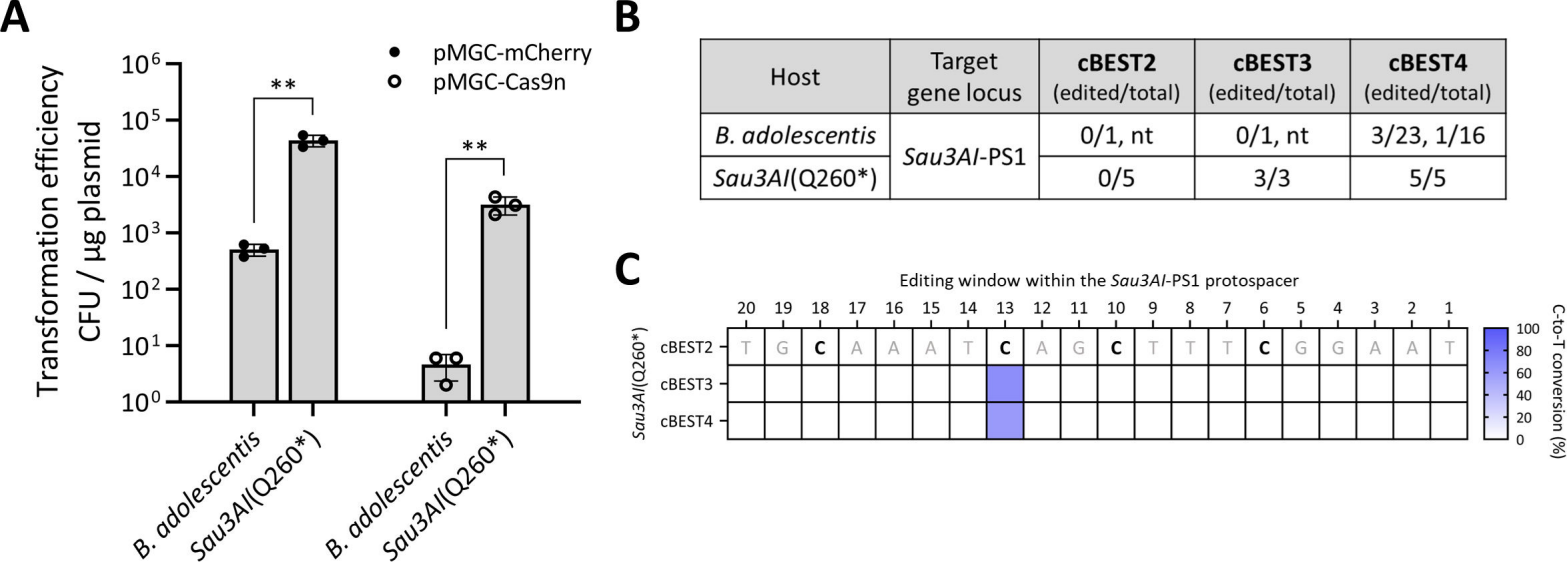
Assessment of REase mutant strain derived from *B. adolescentis* DSM 20083 through transformation and gene editing efficiencies. (**A**) Comparison of transformation efficiencies between wild type and the *Sau3AI*(Q260*) strain using pMGC-mCherry and pMGC-Cas9n plasmids (*n* = 3, biological triplicates; error bars, SEM). **P < 0.01. (**B**) Editing efficiencies were expressed as (number of edited colonies / number of sequenced colonies) of *Sau3AI*-PS1 locus with data from independent transformations separated by commas. For transformations yielding less than five transformants, all transformants were sequenced. (**C**) Editing windows for the three cBEST constructs targeting the *Sau3AI*-PS1 locus in the *Sau3AI*(Q260*) strain. nt, no transformant obtained.

To determine if disruption of RM systems could enhance genome editing efficiencies in another *Bifidobacterium* sp., we compared wild-type *Bifidobacterium adolescentis* DSM 20083 to an RM-disrupted *Sau3AI*(Q260*) strain, which was previously isolated in the lab. As expected for an active REase Sau3AI in *B. adolescentis* DSM 20083, we observed a significant increase in transformation efficiencies using pMGC-mCherry and pMGC-Cas9n plasmids in the RM-disrupted strain (Fig. 3A). In addition, genome editing efficiencies were enhanced in the *Sau3AI*(Q260*) strain compared to the wild-type strain with 100% (5/5) of randomly selected cBEST3 and cBEST4 harboring the desired C-to-T edits within the expected editing window (Fig. 3B and C; Fig. S8), similar to observations in *B. longum* NCIMB 8809.

Overall, these data revealed that native RM systems constitute a major barrier to transformation and genome editing in *Bifidobacterium* and that disruption of RM systems can dramatically enhance genome editing efficiencies in these genetically recalcitrant bacteria.

### Engineered *Bifidobacterium* strains enable streamlined genome editing

**Fig 4:**
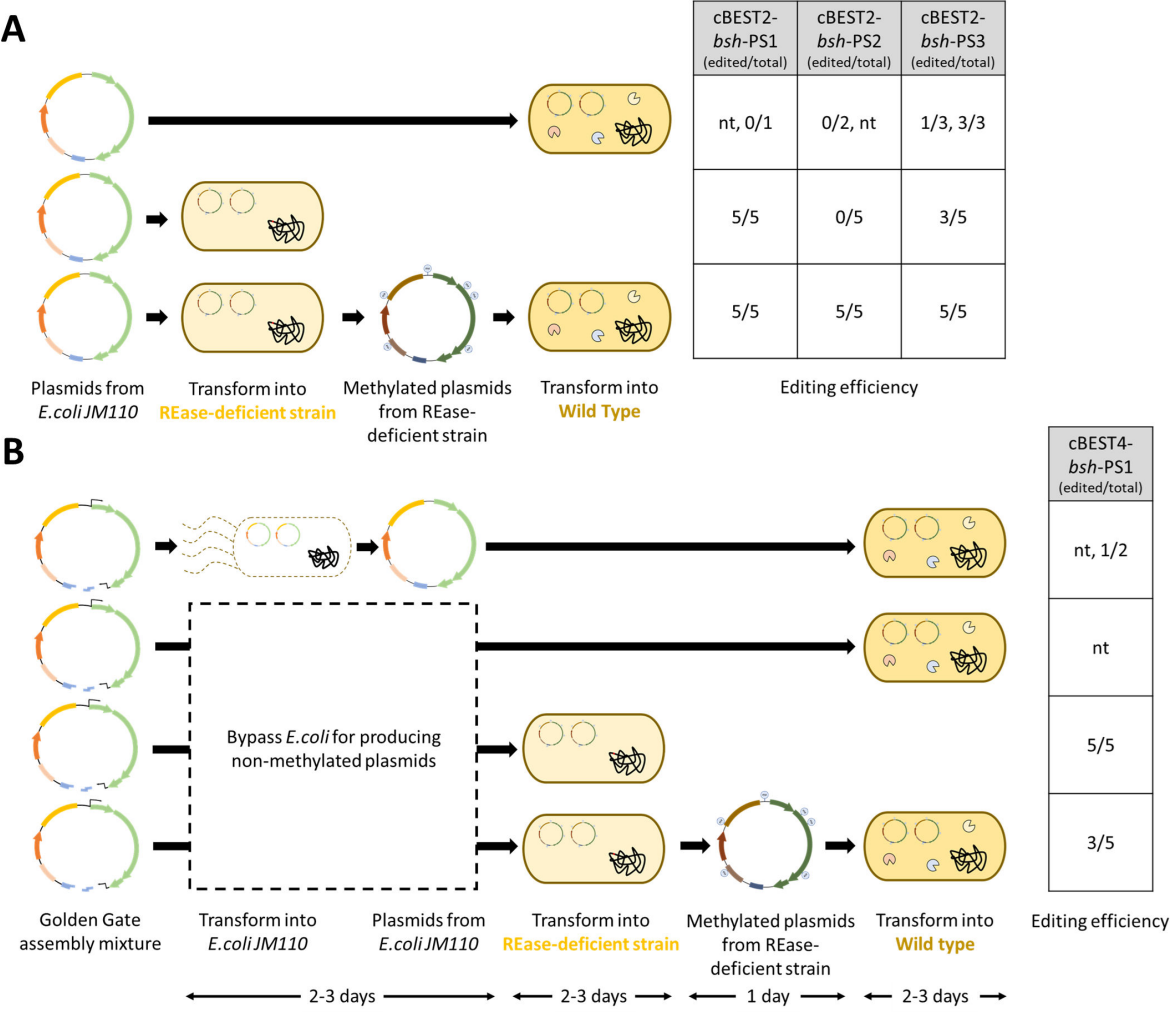
Evaluation of gene editing efficiencies through strategies for RM system evasion and streamlined genome editing. (**A**) Three strategies to evaluate the role of methylated plasmids and REase-deficient strains in evading RM systems. Plasmids were methylated by the *0606*(Q64*)*0983*(W138*)*HsdR*(W29*) strain to match the methylation patterns of the parent host, enabling evasion of RM systems. Non-methylated plasmids were transformed into both *B. longum* NCIMB 8809 wild type and the *0606*(Q64*)*0983*(W138*)*HsdR*(W29*) strain, while the methylated plasmids were only transformed into the wild type. Comparison of editing efficiencies using the cBEST2 constructs containing three unique protospacers (PS1–PS3) within the bile salt hydrolase (*bsh*) gene through the three strategies. (**B**) Strategy for direct methylation and amplification through REase-deficient strains. One-pot Golden Gate assembly mixture for constructing the cBEST4-*bsh*-PS1 plasmid was directly transformed into the *0606*(Q64*)*0983*(W138*)*HsdR*(W29*) strain to evaluate gene editing efficiency. The intact methylated plasmid was then extracted and retransformed into the wild-type *B. longum* NCIMB 8809. Editing efficiencies for each experiment were expressed as (number of edited colonies / number of sequenced colonies) with data from independent transformations separated by commas. For transformations yielding less than five transformants, all transformants were sequenced. nt, no transformants obtained.

The RM-disrupted strains offered an opportunity to bypass the RM systems for highly efficient transformation and genome editing of *Bifidobacterium* spp. By first passaging plasmids through the REase mutant strains, which retained the endogenous machinery to methylate the DNA, we should bypass the native RM systems in the parent strains (Fig. 4A). As expected with successful RM system evasion, transformation of the pMGC-Cas9n plasmid isolated from the *B. longum* NCIMB 8809 *0606*(Q64*)*0983*(W138*)*HsdR*(W29*) and *B. adolescentis* DSM 20083 *Sau3AI*(Q260*) strains into their respective wild-type strains improved transformation efficiencies by more than two to three orders of magnitude compared to the non-methylated plasmid (Fig. S9). In all strains tested, host-methylated plasmids yielded significantly lower numbers of transformants in non-cognate *Bifidobacterium* strains (Fig. S9). The incompatibility of DNA methylation patterns was observed even for strains within the same species, where methylated plasmids isolated from *B. longum* NCIMB 8809 *0606*(Q64*)*0983*(W138*)*HsdR*(W29*) yielded significantly less transformants in *B. longum* DSM 20219 compared to non-methylated plasmids (Fig. S9). Given the inherent diversity of RM systems in the *Bifidobacterium* genus (34), this RM evasion strategy will depend on the availability of cognate REase mutants of *Bifidobacterium* strains of interest.

Given the high transformation and genome editing efficiencies in REase mutant strains, we postulated that circumventing RM systems using host-methylated plasmids will enhance genome editing of wild-type strains. To test this, we sought to disrupt the bile salt hydrolase (*bsh*) gene in *B. longum* NCIMB 8809 due to its critical role in bile acid metabolism. Bile acids serve as key metabolites that govern host fat emulsification, glucose and lipid homeostasis, cholesterol metabolism, and gut microbiota composition (36). Consistent with our previous observations, non-methylated cBEST plasmids yielded very few transformants and, depending on the protospacer used, generally low editing efficiencies in wild-type *B. longum* NCIMB 8809 (Fig. 4A). Similar to our observations in RM-disrupted strains, bypass of native RM systems through the use of host-methylated plasmids greatly increased the number of transformants and gene editing efficiencies, with 100% editing efficiencies achieved independent of the protospacers used (Fig. 4A; Fig. S10).

The high transformation and editing efficiencies attained in the *0606*(Q64*)*0983*(W138*)*HsdR*(W29*) *B. longum* NCIMB 8809 strain prompted us to improve its utility as a model *B. longum* strain by further streamlining the cloning and genome editing process (Fig. 4B). Following *in vitro* Golden Gate assembly of the all-in-one cBEST plasmid, direct transformation of the assembly mix into the *0606*(Q64*)*0983*(W138*)*HsdR*(W29*) *B. longum* NCIMB 8809 strain yielded 100% editing at the target *bsh*-PS1 genomic locus (Fig. 4B; Fig. S11A). Alternatively, prior to plasmid curing, host-methylated cBEST plasmids reisolated from these strains could be used to target the same genomic locus in the wild-type *B. longum* NCIMB 8809 strain (Fig. 4B; Fig. S11B). This streamlined workflow through the *0606*(Q64*)*0983*(W138*)*HsdR*(W29*) *B. longum* NCIMB 8809 strain circumvented the use of *E. coli* as a host for editing plasmid preparation while generating host-methylated editing plasmids and genome-engineered *Bifidobacterium* strains (after plasmid curing) in a single transformation. Notably, no transformants were obtained with this workflow using the parent *B. longum* NCIMB 8809 strain (Fig. 4B). Altogether, these results demonstrated the potential of streamlined genome editing using RM-disrupted *Bifidobacterium* strains.

### cBEST-mediated genome editing enables targeted metabolic perturbations in multiple *Bifidobacterium* spp.

**Fig 5:**
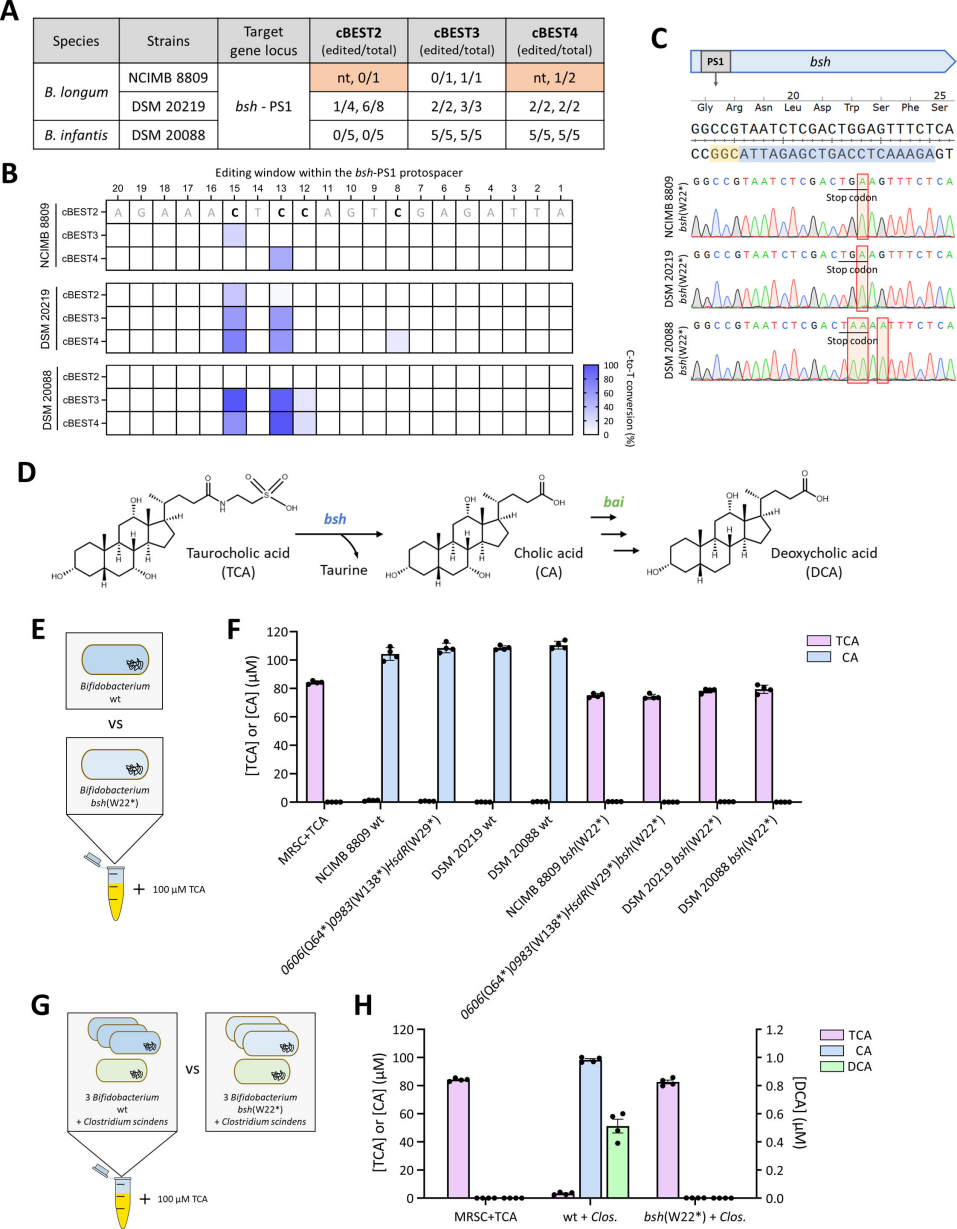
Disruption of a conserved bile acid biosynthetic pathway in different *Bifidobacterium* strains using the same editing plasmid. (**A**) Comparison of editing efficiencies using the cBEST2-*bsh*-PS1, cBEST3-*bsh*-PS1, and cBEST4-*bsh*-PS1 plasmids across *Bifidobacterium* strains NCIMB 8809, DSM 20219, and DSM 20088. Editing efficiencies for each experiment were expressed as (number of edited colonies / number of sequenced colonies) with data from independent transformations separated by commas. For transformations yielding less than five transformants, all transformants were sequenced. Data shaded orange were first presented in Fig. 4A and reproduced here to facilitate comparison between the different constructs. (**B**) Editing window for all tested conditions. (**C**) Successful *bsh*(W22*) strains using the identical cBEST4-*bsh*-PS1 plasmid. Sequencing results confirmed the precise introduction of the stop codon due to successful C-to-T edits (red frames). (**D**) Biosynthesis pathway from taurocholic acid (TCA) to deoxycholic acid (DCA) by gut microbiota. Bile salt hydrolase (*bsh*) deconjugates TCA into cholic acid (CA), and bile acid-inducible operon (*bai*) converts CA into DCA. (**E**) Individual wild-type or *bsh*(W22*) strains cultured in TCA-supplemented MRSC broth for 24 h. (**F**) LC-MS/MS quantification of TCA and CA metabolites in indicated *Bifidobacterium* wild-type and *bsh*(W22*) strains. (**G**) Three *Bifidobacterium* wild-type or *bsh*(W22*) strains with *Clostridium scindens* DSM 5676 (*Clos*.) co-cultures in TCA-supplemented MRSC broth for 24 h. (**H**) LC-MS/MS quantification of TCA, CA, and DCA metabolites in two co-cultures. *n* = 4, biological replicates; error bars, SEM. nt, no transformant obtained.

To determine if cBEST works in *Bifidobacterium* strains other than *B. longum* NCIMB 8809 and *B. adolescentis* DSM 20083, we leveraged the fact that the *bsh* gene is highly conserved in *Bifidobacteria*, including 100% identity at the *bsh*-PS1 target region. As such, we attempted to disrupt *bsh* in *B. infantis* DSM 20088 and *B. longum* DSM 20219 using the same set of cBEST plasmids generated for *B. longum* NCIMB 8809. For direct comparison between strains, unmethylated plasmids isolated from *E. coli* JM110 were used. Varying editing efficiencies of the cBEST constructs in different strains again highlighted the importance of fine-tuning base editor and sgRNA expression (Fig. 5A). Interestingly, we observed a difference in the profile of C-to-T edits within the editing window, depending on the strain and cBEST construct (Fig. 5B). In *B. longum* DSM 20219, the cBEST2-*bsh*-PS1 plasmid yielded C-to-T edits without the introduction of a stop codon, while the cBEST3-*bsh*-PS1 and the cBEST4-*bsh*-PS1 plasmids were able to introduce stop codons with 100% gene editing efficiency (Fig. S12 A, C, and F). For the same cBEST4-*bsh*-PS1 plasmid, the location of the C-to-T edits also consistently differed among *B. longum* NCIMB 8809, *B. longum* DSM 20219, and *B. infantis* DSM 20088 (Fig. 5C; Fig. S12 E through G). It is possible that variations in base editor expression levels and/or diversity in genome methylation patterns could contribute to these strain-specific differences (13).

Since BSH is involved in deconjugation of glycine- or taurine-conjugated bile acids (Fig. 5D), to determine if the *bsh* gene function was disrupted in the *bsh*(W22*) mutants, we quantified the levels of taurocholic acid (TCA), cholic acid (CA), and deoxycholic acid (DCA) levels in TCA-supplemented bacterial cultures (Fig. S13). As expected for bacteria expressing BSH, both wild type and the *0606*(Q64*)*0983*(W138*)*HsdR*(W29*) *B. longum* NCIMB 8809 strains consumed TCA and produced CA when cultured in TCA-supplemented growth medium (Fig. 5F). In contrast, the *bsh*(W22*) strains were unable to metabolize TCA, and CA was undetectable in the *bsh*(W22*) and *0606*(Q64*)*0983*(W138*)*HsdR*(W29*)*bsh*(W22*) cultures (Fig. 5F). Similar observations were obtained with *bsh*-deficient *B. infantis* DSM 20088 and *B. longum* DSM 20219 (Fig. 5F). We further stimulated the production of secondary bile acids *in vitro* through co-culture with *Clostridium scindens* DSM 5676 (Fig. 5G), which possessed the enzymes to convert CA into DCA. As expected, no DCA was detected upon co-culture of the *bsh*-disrupted strains with *C. scindens* DSM 5676 (Fig. 5H), demonstrating the use of portable cBEST-mediated genome editing to achieve specific metabolic perturbations in *Bifidobacteria*.

## DISCUSSION

Here we developed a set of cBEST plasmids for efficient cytosine base editing in *Bifidobacterium* spp. Harboring different promoters, these cBEST plasmids exhibited varying editing efficiencies in different genomic contexts in different strains, highlighting the importance of balancing sgRNA and base editor expression levels. Adding to the limited number of *Bifidobacterium* promoters that can be used for heterologous gene expression (10, 11), here we demonstrated the general functionality of two synthetic promoters, P_tcp830_ and P_kasO__*_, in *Bifidobacteria*. Compared to existing *Bifidobacterium* promoters, these minimally sized (<100 bp) and well-characterized promoters can be engineered to achieve a promoter suite of graded strength or combined with other synthetic modular regulatory elements such as ribosomal binding sites and insulators for predictable control of gene expression in the *Bifidobacterium* genus (23–25). Expanding the collection of regulatory elements in *Bifidobacterium* will benefit the development of synthetic biology tools and rational engineering of metabolic pathways for one of the most important groups of gut commensals and probiotics.

Conventional genetic manipulation of *Bifidobacterium* strains relied heavily on homologous recombination, which required sufficiently high transformation efficiencies and extensive screening for desired recombinants (10, 37, 38). The isolation of desired mutants in seven different genes despite the low number of transformants sequenced highlights the utility of cBEST for genome editing in *Bifidobacteria*, which are typically difficult to transform. Recently, two groups successfully harnessed endogenous CRISPR-Cas systems and homology-directed repair in *Bifidobacterium animalis* subsp. *lactis* DSM 10140 and *Bifidobacterium breve* strains to perform deletions (single gene and large deletions up to 27 kb), single base substitutions, and stop codon insertions (12, 13). For strains lacking characterized CRISPR-Cas systems, exogenous CRISPR/Cas9 systems have been developed for gene deletions in *B. animalis* AR688 (14). An exogenous CRISPR-based cytosine base editor has been reported for *B. lactis* DSM 10140, albeit with high levels (>50%) of inadvertent C-to-A editing (13). Here, using an uracil DNA glycosylase inhibitor-fused base editor, we observed relatively low levels (5 of 146 = 3.4%) of unintended mutations among the 146 edited mutants in *B. longum*, *B. adolescentis*, and *B. infantis*. These inadvertent mutations are unlikely to be a general feature of cBEST since all five mutations—two indels and three C-to-A edits (Fig. S3 and S6)—can be traced to a single editing construct (cBEST2-SpeE-PS1) among the 18 editing constructs used in this study. Additionally, all but one of the C-to-T edits fall within the expected editing window of canonical cytosine base editors using spCas9 previously established in mammals and other bacteria (28, 39, 40). It is encouraging that genome-wide SNP profiling of cBEST off-targets in *Streptomyces* revealed very mild off-target effects, supporting the broad use of cBESTs for genome editing of streptomycete genomes (28). Nonetheless, CRISPR-Cas-mediated genome editing for *Bifidobacteria* is still in its infancy, and its associated genome-wide off-target effects in bifidobacterial genomes remain to be evaluated. Together, these CRISPR tools open up new opportunities for the genetic manipulation of *Bifidobacteria*, including the development of more complex CRISPR-based genome editing strategies involving engineered base editors, prime editors, as well as multiplex editing (15).

The diverse RM systems present a major obstacle to genetic accessibility of the *Bifidobacterium* genus. Here we showed that DNA methylation and RM systems constitute a significant barrier to base editing in *Bifidobacteria*, most likely by limiting the delivery of editing plasmids. At the same time, we demonstrated the opportunities for disrupting or bypassing RM systems for efficient editing of bifidobacterial genomes. Most RM bypass strategies involve mimicking host methylation patterns (41), including *in vitro* methylation with recombinant MTases and *in vivo* methylation by passaging through engineered or related host strains able to match the methylation patterns of the target host strain (42). The *0606*(Q64*)*0983*(W138*)*HsdR*(W29*) *B. longum* NCIMB 8809 and *Sau3AI*(Q260*) *B. adolescentis* DSM 20083 strains will be useful for generating methylated plasmids to bypass RM systems in the wild-type human isolates and will facilitate forward genetic screening approaches to gain better understanding of the genes contributing to *Bifidobacterium* physiology and the health benefits they bring as gut commensals (43). Additionally, the high transformation efficiencies resulting from RM bypass may also facilitate more sophisticated genome editing techniques such as multiplex gene editing (44). Besides mimicking host methylation patterns, a complementary approach known as syngenic DNA, in which RM recognition motifs on plasmids are eliminated (45), has been successful in improving transformation efficiencies in *Staphylococcus aureus* (46). Given the diversity of MTases and methylation profiles across different *Bifidobacterium* species, new generations of RM-silent cBEST plasmids could enable portable genome editing with high editing efficiency in a wider range of genetically recalcitrant strains (46).

For genetically intractable strains of interest, working in a closely related strain for which there are available genetic tools may be a viable strategy. For gut microbes, *Bifidobacterium breve* UCC2003 has been developed as a model strain for the Bifidobacteriaceae family due to its ease of transformation and is being used to explore molecular determinants of host colonization (47, 48). This work introduces the *0606*(Q64*)*0983*(W138*)*HsdR*(W29*) *B. longum* NCIMB 8809 strain as a genetically tractable model for members of the species, which constitute another predominant *Bifidobacterium* spp. of human gut microbiota (49, 50). With transformation efficiencies similar to *B. breve* UCC2003, transposon mutant libraries or CRISPR interference screens are now possible to uncover genotype–phenotype associations in the context of *B. longum* physiology and its host interactions. Additionally, together with cBESTs and one-step streamlined cloning workflow described here, the high genome editing efficiencies in *0606*(Q64*)*0983*(W138*)*HsdR*(W29*) *B. longum* NCIMB 8809 should be useful for rational engineering of additional chassis strains for diverse research and industrial applications. The generation of *metK* and *bsh* knockout strains demonstrated how the cBEST plasmids may be used to achieve targeted metabolic perturbations in *Bifidobacterium*. Utilizing genetically modified *Bifidobacterium* strains with specific metabolic perturbations in appropriate animal models should facilitate more comprehensive investigations into the roles of microbial metabolites in host physiology and disease (36, 51).

In conclusion, we developed a set of cBEST editing plasmids that is portable and functional across multiple *Bifidobacterium* strains. These plasmids can be cured to allow for multiple rounds of genome editing. While fine-tuning of base editor and sgRNA expression could help improve genome editing efficiencies, we showed that evasion of host-specific RM systems is the superior strategy to achieve high editing efficiencies in *Bifidobacterium*. Looking ahead, the ability to efficiently edit and engineer *Bifidobacterium* genomes will undoubtedly give rise to new opportunities for research and applications toward improving human health.

## MATERIALS AND METHODS

### Bacterial strains and culturing

Bacterial strains used in this study are listed in Table S1. *Bifidobacterium* strains and *Clostridium scindens* DSM 5676 were propagated in the Bioresource Collection and Research Center (BCRC) and cultured in NEOGEN Lactobacilli de Man, Rogosa, and Sharpe broth (LOT #US113980G) containing 0.05% wt/vol L-cysteine (MRSC) at 37°C under anaerobic conditions (80% N_2_, 10% CO_2_, 10% H_2_) using Whitley DG250 anaerobic workstation. Bacterial growth curves were carried out in 20 mL MRSC broth inoculated with 400 µL overnight seed cultures over 24 h. Aliquots were removed at indicated time points for OD_600_ measurements using a Metertech Model 6^+^ photometer.

### Construction of Golden Gate-compatible cBEST plasmids

All PCR reactions were performed using KOD Xtreme Hot Start DNA Polymerase (NOVAGEN). DNA oligonucleotides were obtained from Integrated DNA Technologies. All plasmids and primers used in this study are listed in Table S2 and S3, respectively. To facilitate construction of the Golden Gate-compatible cBEST plasmids, *Bbs*I sites were removed from pMGC-mCherry and from the base editor coding sequence (cBEST) of pCRISPR-cBEST (Addgene 125689) using the QuikChange Lightening Multi Site-Directed Mutagenesis Kit (Stratagene, 210513). Additionally, the *EcoR*I site within cBEST was removed. Subsequently, the modified pCRISPR-cBEST was used as a PCR template to amplify cBEST, to which a P_kasO*_ or P3 promoter was added using Golden Gate assembly. Two microliters of Golden Gate assembly reaction was amplified by PCR to obtain P_kasO*_-cBEST and P3-cBEST fragments flanked by *EcoR*I and *Spe*I sites (fragment 1). P_kasO*17__tss_ or P_tcp830_ was added to the Golden Gate-compatible sgRNA cassette from pCRISPomyces-2 by PCR to yield fragment 2 flanked by *Spe*I and *Not*I sites. Fragment 3 contained the pNCC293 origin, and the chloramphenicol resistance cassette was amplified from pMGC-mCherry with flanking *Not*I and *EcoR*I sites. The three fragments were digested using respective restriction enzymes and ligated using T4 DNA ligase (New England Biolabs). All constructs were validated by restriction digest and Sanger sequencing.

### Golden Gate assembly and preparation of cBEST plasmids

Complementary oligonucleotide pairs (4 µM each) were annealed by heating at 95°C for 5 min and then gradually cooled to 4°C (0.1 °C/s) in annealing buffer (10 mM Tris–HCl, pH 8.5, and 50 mM NaCl) prior to phosphorylation using T4 polynucleotide kinase (New England Biolabs) at 37°C for 1 h in a thermocycler. Annealed protospacers were inserted into cBEST plasmids via *Bbs*I-mediated Golden Gate assembly: cycle between (37°C, 10 min → 16°C, 10 min) × 10 cycles followed by 50°C, 5 min, and 65°C, 20 min. The Golden Gate assembly reaction was transformed into Z-competent DH10β *E. coli*, and cells were plated onto X-gal/IPTG/chloramphenicol lysogeny broth plates for blue-white screening. White colonies were picked for miniprep and verified using Sanger sequencing. Verified plasmids were retransformed, and JM110 *E. coli* was prepared to obtain non-methylated plasmids to be electroporated into *Bifidobacterium* spp.

### Protospacer design for gene knockout

The protospacer design followed the previous work (28). Briefly, we identified Gln codons (CAA and CAG) on the forward strand or Trp (AGG) codons on the reverse strand within the theoretical editing window, which is situated 11–17 nucleotides upstream from PAM (NGG) (28). Next, a BLAST search of 12nt + NGG was conducted to ensure only a single perfect match in the whole genome. Usually, two to three protospacers were selected per gene to ensure successful gene editing. The protospacers used in this study are listed in Table S4.

### Electroporation of *Bifidobacterium* strains

Culture medium was first optimized for *B. longum* NCIMB 8809 (Fig. S1). Overnight seed cultures in MRSC broth were diluted 30-fold in MRSC broth supplemented with 0.2 M sucrose and 0.2 M NaCl and incubated anaerobically until OD_600_ reached 0.3–0.4 (52). Other *Bifidobacterium* spp. were cultured in MRSC broth supplemented with 0.2 M sucrose. After bacterial culturing, bacteria were chilled on ice for 15 min, washed with cold 10% glycerol twice, and resuspended in 1/75 of the original culture volume of 10% glycerol. For plasmid transformation, 0.1 mL bacteria were mixed with plasmids (1.5 µg non-methylated cBEST plasmids, 500 ng non-methylated pMGC-mcherry or pMGC-Cas9n plasmids, or 250 ng host-methylated plasmids). For direct transformation of the Golden Gate assembly reaction, 5 µL of the assembly reaction was added to 0.1 mL of bacteria. Mixtures were chilled on ice for 15 min. Upon transfer to a pre-chilled 0.1 cm cuvette, cells were electroporated at 1.5 kV/cm for 5 ms using MicroPulser Electroporator (Bio-Rad) and recovered immediately with 0.9 mL MRSC broth for 2.5 h at 37°C anaerobically. The bacteria were plated on MRSC agar containing 10 µg/mL of chloramphenicol and incubated at 37°C anaerobically for 2–3 days.

### Determination of transformation efficiencies

Transformation efficiencies were determined by independent electroporation experiments with the plasmid quantities described above. CFUs were counted on chloramphenicol resistance plates following appropriate dilutions. Transformation efficiencies were finally calculated as CFU per microgram of plasmid.

### Validation of genome editing and determination of genome editing efficiencies as well as editing windows

Editing plasmids (cBEST2, cBEST3, or cBEST4) were transformed into *Bifidobacterium* spp. and plated on chloramphenicol selection MRSC agar plates. For editing of wild-type strains, all transformants were screened by colony PCR of the target genomic loci followed by Sanger sequencing of the PCR products. For editing in REase-deficient strains and *B. infantis* DSM 20088 or using methylated editing plasmids, five colonies from each plate were randomly picked for colony PCR and sequencing due to their high transformation efficiencies (>10^3^ CFU/µg plasmid). Editing efficiencies were calculated as (number of edited colonies / number of sequenced colonies). “nt” means no transformants were obtained. For editing windows, sequences of protospacers with position numbering are displayed with PAM at positions 0 to −2. The conversion rate for each shaded nucleotide was calculated as (number of C-to-T edits / number of sequenced colonies). Primers used for PCR and Sanger sequencing are listed in Table S3.

### Plasmid curing from *Bifidobacterium* transformants

To cure base editing plasmids from *Bifidobacterium* transformants, chloramphenicol-resistant colonies were streaked onto non-selective MRSC agar plates. Every 2 days, single colonies were restreaked onto new non-selective MRSC plates. After five successive passages, bacteria that were able to grow on non-selective medium but not on chloramphenicol selection plates were used for functional validation and studies. Typically, after five passages on non-selective media, all of the colonies screened were cured of their plasmids, as confirmed by colony PCR (Fig. S5F).

### mCherry reporter assay for promoter screening and characterization

Chloramphenicol-resistant *Bifidobacterium* transformants containing plasmids driving mCherry expression from indicated promoters were inoculated into MRSC broth supplemented with 10 µg/mL chloramphenicol. Overnight cultures were diluted 1:50 into 1 mL MRSC broth with 10 µg/mL chloramphenicol and grown anaerobically at 37°C for 5 or 24 h to obtain exponential- or stationary-phase cultures, respectively. Cells were harvested, washed with phosphate-buffered saline (PBS) twice, and resuspended in 400 µL PBS. Both mCherry fluorescence (554 nm/610 nm) and culture absorbance (600 nm) were measured using Synergy H1 Microplate Reader from BioTek. Relative fluorescence unit was defined as the ratio of fluorescence to absorbance. Three independent biological repeats were performed for mCherry reporter assay experiments. For each biological repeat, three colonies were picked, and fluorescence results were averaged. Sequences of promoters used in this study are listed in Table S5.

### Targeted LC-MS/MS analysis of methionine-derived metabolites

Overnight liquid cultures were plated on a non-selective MRSC agar plate. After 18 h incubation, bacteria were scraped into Eppendorf tubes and measured for wet weight. Six hundred microliters of 80% MeOH was added to each sample, followed by ultrasonication homogenization (25 W, 20 kHz, 40 s) and centrifugation at 13,000 rpm for 10 min at 4°C. The supernatant (50 µL) was mixed with 50 µL internal standards (50 ppb methionine-13C,d3 and 50 ppb MTA-d3) and transferred to autosampler vials for quantitative LC-QqQ analysis. Quantitative LC-MS/MS analysis was performed by an ExionLC AC system coupled to a SCIEX Triple Quad 5500. Three microliters of the sample was injected and separated using an Acquity UPLC BSH C18 column (2.1 × 100 mm, 1.7 µm) at 40°C. Mobile phase A was 0.1% formic acid in deionized water, while mobile phase B was 0.1% formic acid in acetonitrile. The elution gradient for separation was as follows: 5% B for 1 min, increased linearly to 95% B at 5 min, held at 95% B for 3 min, decreased linearly to 5% B at 9 min and held for another 3 min. The multiple reaction monitoring method for individual compounds is listed in Fig. S14. The peak area of L-methionine was normalized to its internal standard, and SAM was normalized to the MTA-d3 internal standard. The multiple reaction monitoring method for individual compounds is detailed in Fig. S14.

### Targeted LC-MS/MS quantification for bile acid production

Ten microliters of overnight *Bifidobacterium* cultures were inoculated into 490 µL MRSC broth supplemented with 100 µM TCA. For bacterial co-culture experiments, three *Bifidobacterium* strains and *Clostridium scindens* DSM 5676 cultures were mixed overnight at the ratio of 1:1:1:3 and inoculated into TCA-supplemented MRSC broth. After 24 h incubation, 600 µL of ice-cold 100% LC-grade methanol spiked in two ppm cholic acid-d4 was mixed with 150 µL cultures thoroughly. Samples were homogenized by ultrasonication (25 W, 20 kHz) for 1 min and then centrifuged at 13,000 rpm for 10 min at 4°C. Supernatants were further diluted five times into 80% LC-grade methanol and transferred to autosampler vials for LC-QqQ analysis.

Quantitative LC-MS/MS was performed by an ExionLC AC system coupled to a SCIEX Triple Quad 5500. Three microliters of sample was injected and separated using an Acquity UPLC BSH C18 column (2.1 × 100 mm, 1.7 µm) at 40°C. Mobile phase A was 1 mM ammonium acetate in deionized water, while mobile phase B was 95% acetonitrile with 5% A. The elution gradient for separation was as follows: 30% B for 1 min, increased linearly to 100% B at 8 min, held at 100% B for 3 min, and decreased linearly to 30% B at 12 min and held for another 3 min. The multiple reaction monitoring method for individual compounds and their calibration curves are listed in Fig. S13. Standard mixtures with a concentration range from 1 to 200 µM were prepared following the same protocol.

### Statistical analysis

All statistical analyses were performed in GraphPad Prism version 9. For the analysis of the mCherry expression level, unpaired two-tailed Student’s *t*-test was conducted for comparison between exponential and stationary cultures. For the analysis of transformation efficiency, one-way analysis of variance (ANOVA) followed by Tukey’s post hoc test was employed for multiple comparisons, or unpaired two-tailed Student’s *t*-test was used for comparison between two groups. For analysis of methionine-derived metabolites, one-way ANOVA followed by Dunnett’s multiple comparisons was used to compare with the *B. longum* NCIMB 8809 strain. Asterisks represent significant differences in *P* values (*P < 0.05, **P < 0.01, ***P < 0.001, ****P < 0.0001), and ns represents no statistical significance.

## Data Availability

cBEST2 (plasmid #234658), cBEST3 (plasmid #234659), and cBEST4 (plasmid #234660) are available from Addgene. Other materials, e.g., the *Bifidobacterium* strains, will be made available by the authors upon request.

## SUPPLEMENTAL MATERIAL

Supplemental material aem.01985-24-s0001.pdf Tables S1 to S7, Figures S1 to S14, and supplemental methods.

ASM does not own the copyrights to Supplemental Material that may be linked to, or accessed through, an article. The authors have granted ASM a non-exclusive, world-wide license to publish the Supplemental Material files. Please contact the corresponding author directly for reuse.
