# Supplementary material for: Highly efficient CRISPR-Cas9 base editing in Bifidobacterium with bypass of restriction modification systems
Source: Appl Environ Microbiol. 2025 Mar 10;91(4):e01985-24. doi: 10.1128/aem.01985-24 (PMC12016496; doi:10.1128/aem.01985-24)
Supplement: Supplemental material — Tables S1 to S7, Figures S1 to S14, and supplemental methods. [file aem.01985-24-s0001.pdf]

Supplementary Information for

Highly Efficient DSB-Free CRISPR-Cas9 Base Editing in *Bifidobacterium* with Bypass of Restriction Modification Systems

Hung-Chun Lin,[1,#] Wan-Chi Hsiao,[2,3,#] Ya-Chen Hsu,[1] Meng-Chieh Lin,[1] Cheng-Chih Hsu[1,4,*] and Mingzi M. Zhang[3,*]

[1]Department of Chemistry, National Taiwan University, Taipei, 10617, Taiwan

[2]Institute of Biotechnology, National Tsing Hua University, Hsinchu, 30013, Taiwan

[3]Institute of Molecular and Genomic Medicine, National Health Research Institutes, Miaoli, 35053, Taiwan

[d]Leeuwenhoek Laboratories Co. Ltd, Taipei, 10617, Taiwan

Joint Authors

* Corresponding Authors

Cheng-Chih Hsu, Email: ccrhsu@ntu.edu.tw

Mingzi M. Zhang, Email: zhangmz@nhri.edu.tw

Supplementary Tables **Pages S2-S9**

Supplementary Figures **Pages S10-S25**

Supplementary Methods **Page S26**

Table S1. Bacteria strains used in this study.

Strains	Description	source
E.coli		
DH10β	For routine plasmids maintenance and cloning	NEB
JM110	For unmethylated (Dam- Dcm-) plasmid expression	ATCC
B. longum subsp. longum		
NCIMB 8809	isolate from Nursling stools	BCRC
SpeE (Q198*)	NCIMB 8809 mutant in *SpeE* (Q198*) using cBEST2-*SpeE* -PS2 plasmid	This study
SpeE (Q117*)	NCIMB 8809 mutant in *SpeE* (Q117*) using cBEST2-*SpeE* -PS1 plasmid	This study
HsdR (W29*)	NCIMB 8809 mutant in *HsdR* (W29*) using methylated cBEST2-*HsdR* -PS1 plasmid	This study
0983 (W138*)	NCIMB 8809 mutant in *EcoRII_0983* (W138*) using cBEST2-*0983* -PS1 plasmid	This study
0606 (Q64*)	NCIMB 8809 mutant in *EcoRII_0606* (Q64*) using cBEST2-*0606* -PS2 plasmid	This study
0606 (Q64*)*0983* (W138*)	*0606* (Q64*) mutant in *EcoRII_0983* (W138*) using cBEST2-*0983* -PS1 plasmid	This study
0606 (Q64*)*0983* (W138*)*HsdR* (W29*)	*0606* (Q64*)*0983* (W138*) mutant in *HsdR* (W29*) using cBEST2-*HsdR* -PS1 plasmid	This study
0983 (W138*)*MetK* (Q134*)	*0983* (W138*) mutant in *MetK* (Q134*) using cBEST4-*MetK* -PS1 plasmid	This study
0606 (Q64*)*MetK* (Q134*)	*0606* (Q64*) mutant in *MetK* (Q134*) using cBEST4-*MetK* -PS1 plasmid	This study
0606 (Q64*)*0983* (W138*)*MetK* (Q134*)	*0606* (Q64*)*0983* (W138*) mutant in *MetK* (Q134*) using cBEST4-*MetK* -PS1 plasmid	This study
0606 (Q64*)*0983* (W138*)*HsdR* (W29*)*MetK* (Q134*)	*0606(Q64*)0983(W138*)HsdR(W29*)* mutant in *MetK* (Q134*) using cBEST4-*MetK* -PS1 plasmid	This study
bsh (W22*)	NCIMB 8809 mutant in *bsh* (W22*) using methylated cBEST2-*bsh* -PS1 plasmid	This study
0606 (Q64*)*0983* (W138*)*HsdR* (W29*)*bsh* (W22*)	*0606* (Q64*)*0983* (W138*)*HsdR* (W29*) mutant in *bsh* (W22*) using cBEST2-*bsh* -PS1 plasmid	This study
DSM 20219	type strain, isolate from adult intestine	BCRC
bsh (W22*)	DSM 20219 mutant in *bsh* (W22*) using cBEST4-*bsh* -PS1 plasmid	This study
B. adolescentis		
DSM 20083	type strain, isolate from adult intestine	BCRC
Sau3AI (Q260*)	spontaneous mutant in *Sau3AI* (Q260*)	This study
B. longum subsp. infantis		
DSM 20088	type strain, isolate from infant intestine	BCRC
bsh (W22*)	DSM 20088 mutant in *bsh* (W22*) using cBEST4-*bsh* -PS1 plasmid	This study
Clostridium scindens		
DSM 5676	type strain, isolate from human faeces	BCRC

Table S2. Plasmids used in this study.

Plasmids	Description	References and Sources
pMGC-mcherry	Evaluate transformation efficiencies in *Bifidobacterium* spp.	(15)
pMGC-Cas9n		This study
P6-mCherry	Examine the portability of promoters in *Bifidobacterium* spp.	This study
P3-mcherry		This study
$P_{kasO*17}$-mcherry		This study
P_{kasO*}-mcherry		This study
P_{tcp830}-mcherry		This study
cBEST2	Empty vector for genome editing in *Bifidobacterium* spp.	This study
cBEST3		This study
cBEST4		This study
pCRISPomyces-2	For cBEST plasmids construction, base editor with spCas9n (D10A) fused to APOBEC1 and UGI	(25)
pCRISPR-cBEST	For cBEST plasmids construction, a cytidine deaminase base base editor	(25)

Table S3. Primers used in this study.

Primer names	Sequence(5'-3')	Description
P6-F	TACGACTCACTATAGGGCGAATTGGCCCTGCAGGCGGAAGTCA	PCR P6 promoter
P6-R	TATCCTCCTCGCCCTTGCTCACCATTACGTCTCCGTCGTCTACTCGAGCT	
P3-F	TACGACTCACTATAGGGCGAATTGGCTGCTCCTTCGGTCGGACGT	PCR P3 promoter
P3-L	TATCCTCCTCGCCCTTGCTCACCATGCGTATCCCCTTTCAGATACTCGC	
tcp830-F	TACGACTCACTATAGGGCGAATTGCTGTTGGCTACTCTATCATTGATAG TGGTAGGATCCCTATCAGTGATAGAGATATC	PCR tcp830 promoter
tcp830-R	TATCCTCCTCGCCCTTGCTCACCATATGTCCGCTCCCTTCTGATATCTCTA TCACTGATAGGGATC	
kasop-F	TACGACTCACTATAGGGCGAATTGTGTTCACATTCGAACGGTCTCTGCTT TGACAACATGCTGTGCGGTGTTGTAAAGTCGTGGC	PCR _kasO*_ promoters
kasop17-F	TACGACTCACTATAGGGCGAATTGTGTTCACATTCGAACGGTCTCTGCTT TGACAACATGCTGTGCGGTGTGTAAAGTCGTGGC	PCR _kasO*_17 promoters
kasop-R	TATCCTCCTCGCCCTTGCTCACCATAACTCCCCCAGTCCTGCACGCTGTCG TATTCTCCTGGCCACGACTTTACAACACCG	PCR _kasO*_ and _kasO*_17 promoters
mcherry-R	TTGGTCACCTTCAGCTTGG	sequencing of pMGC-mcherry
Pgap-F	GAGCCTCTCTGACCTGTTCG	
pMGC-seq	GAATGCCTCGCACGAATCG	
longum core-F	TTTGAATTCTGGAGCTCCAGCTTTTGTTC	for construction of the cBEST2, cBEST3 and cBEST4
longum core-R	AAAGCGGCCGCATCCCATTATGCTTTGGCAGT	
PkasO*-F	AAAAGAATTCTGTTCACATTCGAACGGTCT	PCR _kasO*_ promoter for cBEST2 construction
PkasO*-R	TGAAGACGGAGGACATAACTCCCCCAGTCCTGCACG	
Promoter P3-F	AAAAGAATTCGCTGCTCCTTCGGTCGGACG	PCR P3 promoter for cBEST3 and cBEST4 construction
Promoter P3-R	TGAAGACGGAGGACATGCGTATCCCCTTTCAGATAC	
Cas9-F	TGAAGACTGTCCTCCGAGACCGGCCCC	PCR cas9 for cBEST2, cBEST3 and cBEST4 construction
Cas9-R	AAAACTAGTCGAATATATCGGTTATGCGTGG	
PkasO*17tss-F	AAAACTAGTTGTTCACATTCGAACGGTCTCTGCTTTGACAACATGCTGT GCGGTGTGTAAAGTCGTGGCCAAAGTCTTCTCAGCCGCTACAG	for construction of the cBEST2 and cBEST3
Ptcp830-R	AAAACTAGTCTGTTGGCTACTCTATCATTGATAGTGGTAGGATCCCTAT CAGTGATAGAGATAAGTCTTCTCAGCCGCTACAG	for construction of the cBEST4
ori-sgRNA-R	TTTGCGGCCGCTTGAGATCCTTTTTTTCTGCGC	for construction of the cBEST2, cBEST3 and cBEST4
mut1 of BbsI/cBEST	CACGAGTTCGAGGTGTTCTTCGACCCGCG	remove BbsI site in pCRISPR-cBEST
mut2 of BbsI/cBEST	GCAGGTCAACATCGTGAAGAAAACCGAGGTGCA	
mut3 of EcoRI/cBEST	CGGGTACCGAGCTCGGATTCCCCAGATCTAA	remove EcoRI site in pCRISPR-cBEST
mut BbsI	CGCAGGCAAATCCTGCTCTTCTTCCCCAATG	BbsI site mutagenesis in pGMC-mcherry
mut NseI to NsiI	CTCTAGATAATGAGACAGAATTATGATGATCATGCATCAACTAACGGGG CAGGTT	swap NdeI for NsiI in pGMC-mcherry
pBR322ori-F	GGGAAACGCCTGGTATCTTT	sequencing of cBEST
SpeI-_kasOP17tss_-sgRNA-F	AAAACTAGTTGTTCACATTCGAACGGTCT	
SpeI-_tcp830_-sgRNA-F	AAAACTAGTCTGTTGGCTACTCTATCATTGA	
APO_EcoRI site	TGTGGCGCCAGATGGAGTG	
CAM-NotI site	CTCTTTTCTCTTCCAATTGT	

Primer names	Sequence(5'-3')	Description
kas-*SpeE* -PS1-F	gccaTGACCAGAAGATCACCGATC	
kas-*SpeE* -PS2-F	gccaCGGCCAAGGTTCGATCAACG	
kas-*SpeE* -PS3-F	gccaGGTTTCCCAGAACGCGAAGC	
kas-*SpeE* -PS4-F	gccATGGACTCGCCCTCATAGGCC	
tcp-*SpeE* -PS1-F	agatTGACCAGAAGATCACCGATC	
tcp-*SpeE* -PS2-F	agatCGGCCAAGGTTCGATCAACG	
tcp-*SpeE* -PS3-F	agatGGTTTCCCAGAACGCGAAGC	
tcp-*SpeE* -PS4-F	agatTGGACTCGCCCTCATAGGCC	*SpeE* protospacers and construction validation
SpeE -PS1-R	aaacGATCGGTGATCTTCTGGTCA	
SpeE -PS2-R	aaacCGTTGATCGAACCTTGGCCG	
SpeE -PS3-R	aaacGCTTCGCGTTCTGGGAAACC	
SpeE -PS4-R	aaacGGCCTATGAGGGCGAGTCCA	
SpeE- PCR-F	CATGTCCGCTGATCTACTTCATCTCG	
SpeE -PCR-R	TTGTCGTCGGTGAGAATAGTCGACG	
SpeE- Seq	TCTTGGCGAACAGTTCACGGT	
kas-*Sau3AI* -PS1-F	gccaTGCAAATCAGCTTTCGGAAT	
tcp-*Sau3AI* -PS1-F	agatTGCAAATCAGCTTTCGGAAT	
Sau3AI -PS1-R	aaacATTCCGAAAGCTGATTTGCA	*Sau3AI* protospacers and construction validation
Sau3AI -PCR-F	CATGGTGTACTACTACGATGACCGC	
Sau3AI -PCR-R	CCAGGAAGTAAATTGGTTCCTCGTCC	
Sau3AI -Seq	CCTCCAACAAGACGGTCATTACCG	
kas-*bsh* -PS1-F	gccaAGAAACTCCAGTCGAGATTA	
kas-*bsh* -PS2-F	gccaGCTCGTCTCCCAGATTGTGC	
kas-*bsh* -PS3-F	gccaCTCGTCTCCCAGATTGTGCC	
tcp-*bsh*- PS1-F	agatAGAAACTCCAGTCGAGATTA	
tcp-*bsh*- PS2-F	agatGCTCGTCTCCCAGATTGTGC	
tcp-*bsh*- PS3-F	agatCTCGTCTCCCAGATTGTGCC	
bsh -PS1-R	aaacTAATCTCGACTGGAGTTTCT	*bsh* protospacers and construction validation
bsh -PS2-R	aaacGCACAATCTGGGAGACGAGC	
bsh -PS3-R	aaacGGCACAATCTGGGAGACGAG	
bsh -PCR-F	GTGCAAGCCAACCAAAGCGATGG	
bsh- PCR-R	CGTTGCTGACGCACATGTAGTTGC	
bsh -Seq	GCACATCGACGTCATCATGATGCAC	
kas-*HsdR* -PS1-F	gccaTCGTACCATTCCTCGTTCAG	
HsdR -PS1-R	aaacCTGAACGAGGAATGGTACGA	
HsdR -PCR-F	ATGGCTGTTTCCAAAGTCGAATCTCG	*HsdR* protospacer and construction validation
HsdR- PCR-R	GCCGTCAATCATGGTGCTCCAT	
HsdR -Seq	CACGAACATGCTCGGTATCTGCTTC	
kas-*0606* -PS2-F	gccAGACTTTCAGATGCAGCCTCA	
0606 -PS2-R	aaacTGAGGCTGCATCTGAAAGTC	
0606 -PCR-F	TGCCAGACTCAACACTCCTGTAG	*EcoRII_0606* protospacer and construction validation
0606 -PCR-R	ATAGCTGCGCTTGTCACCATCC	
0606 -Seq	AACAATCTCCCGGTACGGCA	
0983 -PS1-F	gccAGTACCATGTGGTTTCAAGGT	
0983 -PS1-R	aaacACCTTGAAACCACATGGTAC	
0983 -PCR-F	ATGCAGGCGGATGGAGAAGA	*EcoRII_0983* protospacer and construction validation
0983 -PCR-R	CAGGAAATCGGGTTTCTTCCCG	
0983 -Seq	GTATTCGATCACCACGTCGTC	
CmR-PCR-F	CAGTCATTAGGCCTATCTGACAATTC	for confirmation of editing plasmid
CmR-PCR-R	GCAGTTTATTCTTGACATGTAGTGAGG	
tcp-*MetK* -PS1-F	agatGGCGATCAGGGCGTGATGTT	
MetK -PS1-R	aaacAACATCACGCCCTGATCGCC	
MetK -PCR-F	GTTCCTGGTCTTCGGCGAAGTCAC	*MetK* protospacer and construction validation
MetK -PCR-R	CGTAGGTGTCCACGATGATCTTGCGG	
MetK -Seq	ATATTGCGACGTGCAGTCCAAGGTC	

Table S4. Protospacers used in this study.

Bacterial strain (accession number)	Target gene	Genbank code	Protospacer code	Sequence (5'-3')	PAM (NGG)
B. longum NCIMB 8809 (CP011964.1)	*SpeE*	ALO72771.1	PS1	TGACCAGAAGATCACCGATC	TGG
			PS2	CGGCCAAGGTTCGATCAACG	AGG
			PS3	GGTTTCCCAGAACGCGAAGC	CGG
			PS4	TGGACTCGCCCTCATAGGCC	AGG
	EcoRII_0606	ALO72279.1	PS2	GACTTTCAGATGCAGCCTCA	GGG
	EcoRII_0983	ALO72654.1	PS1	GTACCATGTGGTTTCAAGGT	CGG
	HsdR	ALO73026.1	PS1	TCGTACCATTCCTCGTTCAG	TGG
	MetK	ALO73020.1	PS1	GGCGATCAGGGCGTGATGTT	CGG
	bsh	ALO72466.1	PS1	AGAAACTCCAGTCGAGATTA	CGG
			PS2	GCTCGTCTCCCAGATTGTGC	CGG
			PS3	CTCGTCTCCCAGATTGTGCC	GGG
B. adolescentis DSM 20083 (AP009256.1)	*Sau3AI*	BAF40013.1	PS1	TGCAAATCAGCTTTCGGAAT	CGG
B. infantis DSM 20088 (CP001095.1)	*bsh*	ACJ52536.1	PS1	AGAAACTCCAGTCGAGATTA	CGG
B. longum DSM 20219 (-)	*bsh*	-	PS1	AGAAACTCCAGTCGAGATTA	CGG

Table S5. Promoters used in this study for heterologous expression.

Name	Length (bp)	Sequence (5'-3')
P_{tcp830}	53	CTGTTGGCTACTCTATCATTGATAGTGGTAGGATCCCTATCAGTGATAGAGAT
$P_{kasO*\,17tss}$	62	TGTTCACATTCGAACGGTCTCTGCTTTGACAACATGCTGTGCGGTGTGTAAAGTCGTGGCCA
$P_{kasO*\,17}$	96	TGTTCACATTCGAACGGTCTCTGCTTTGACAACATGCTGTGCGGTGTGTAAAGTCGTGGCCA<br>GGAGAATACGACAGCGTGCAGGACTGGGGGAGTT
P_{kasO*}	97	TGTTCACATTCGAACGGTCTCTGCTTTGACAACATGCTGTGCGGTGTTGTAAAGTCGTGGCCA<br>GGAGAATACGACAGCGTGCAGGACTGGGGGAGTT
P_{gap}	191	GCGGAATGCCTCGCACGAATCGCCGCAGGCTGTACAGACATATTTGTTAGCGTTAACGAAAT<br>ATGGCCGTTTTATGCTCAAAGCAAGCGCGACACCGTTGCTCTAGTACAGACGGCGCATTACAG<br>TAGACACTGTTGGTAAACAAAGGCCATAGCGCATCCATGCGCAAACGGTCTACCCTACAAAGG<br>GAG
P3	285	GCTGCTCCTTCGGTCGGACGTGCGTCTACGGGCACCTTACCGCAGCCGTCGGCTGTGCGACA<br>CGGACGGATCGGGCGAACTGGCCGATGCTGGGAGAAGCGCGCTGCTGTACGGCGCGCACC<br>GGGTGCGGAGCCCCTCGGCGAGCGGTGTGAAACTTCTGTGAATGGCCTGTTCGGTTGCTTTT<br>TTTATACGGCTGCCAGATAAGGCTTGCAGCATCTGGGCGGCTACCGCTATGATCGGGGCGTT<br>CCTGCAATTCTTAGTGCGAGTATCTGAAAGGGGATACGC
P6	302	GCCCTGCAGGCGGAAGTCAGGTAGACACGACTTCCGCTAGTCCTTGCAAGGTCTGCTGACGT<br>GAGGCGGGGCGGTCGTTTTTGACCGCCCTGCCTTCGTCATGTAGGCTCGCTCGCTGTGCCTG<br>GCGTGTCATCAGACGCCCAGGTCCCGGTGCCGTGAGGCCCGGGCCATCGAGCCGGTGGTAC<br>GTGGCTGCGGTCCCCTTGTGAGGGCTGCGCGCCGTGTGCTGTCCGGCGCGCACAGCCTTGAA<br>TCCACCCGCGGGGGCCGGCCGGTCTCCGTGAGCTCGAGTAGACGACGGAGACGTA

Table S6. Promoters used to express Cas9n fusion protein and sgRNA in cBEST editing plasmids.

	cBEST2	cBEST3	cBEST4
**Cas9n fusion**	P_{kasO*}	P3	P3
**sgRNA**	$P_{kasO*17tss}$	$P_{kasO*17tss}$	P_{tcp830}

Table S7. List of RM systems in used *Bifidobacterium* strains searched from REBASE. REase and MTase are listed in gene name or gene number with the RM type and predicted recognition motif. Number of predicted recognition motifs on both pMGC-mCherry and pMGC-Cas9n plasmids for each RM system. There is no available whole genome sequence for *B. longum* DSM 20219.

Strain	REase	MTase	RM type	Predicted recognition motif	Sites on pMGC-mCherry	Sites on pMGC-Cas9n
B. longum NCIMB 8809	*HsdR*	*HsdM*	I	5'-GATN$_5$TGCC-3'	0	3
	EcoRII (0606, 0983)	*0607*	II	5'-CCWGG-3'	10	34
	-	*0958*	II	5'-TCGGCCGA-3'	0	0
B. adolescentis DSM 20083	*Sau3AI, 1229*	*1233*	II	5'-GATC-3'	15	56
	Rkpn2KI, 1281	*1283*	II	5'-CCNGG-3'	18	55
B. infantis DSM 20088	*0091*	*0092*	II	5'-GGCGCC-3'	2	7
	289R	*289M*	II	5'-CTGCAG-3'	2	3
	1146	*1145*	II	5'-GTCGAC-3'	1	7
	-	*1196*	II	5'-GAATTC-3'	1	1
	-	*1215, 1346*	II	5'-AAGCTT-3'	1	0
	1324	*1324*	II	5'-GAGGAC-3'	6	8

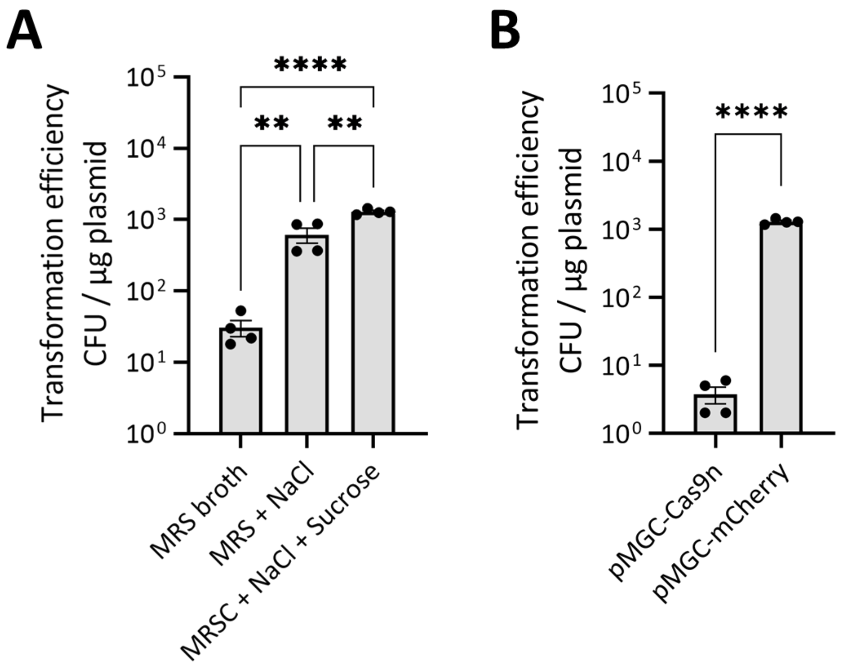

Figure S1. Method optimization in competent cell preparation. (A) Medium optimization for competent cell preparation via pMGC-mCherry. (n=4, biological replicates; error bars, s.e.m.; **P<0.01, ****P<0.0001) (B) Transformation efficiencies of pMGC-mCherry and pMGC-Cas9n plasmids using the optimized electroporation protocol. (n=4, biological replicates; error bars, s.e.m.; ****P<0.0001)

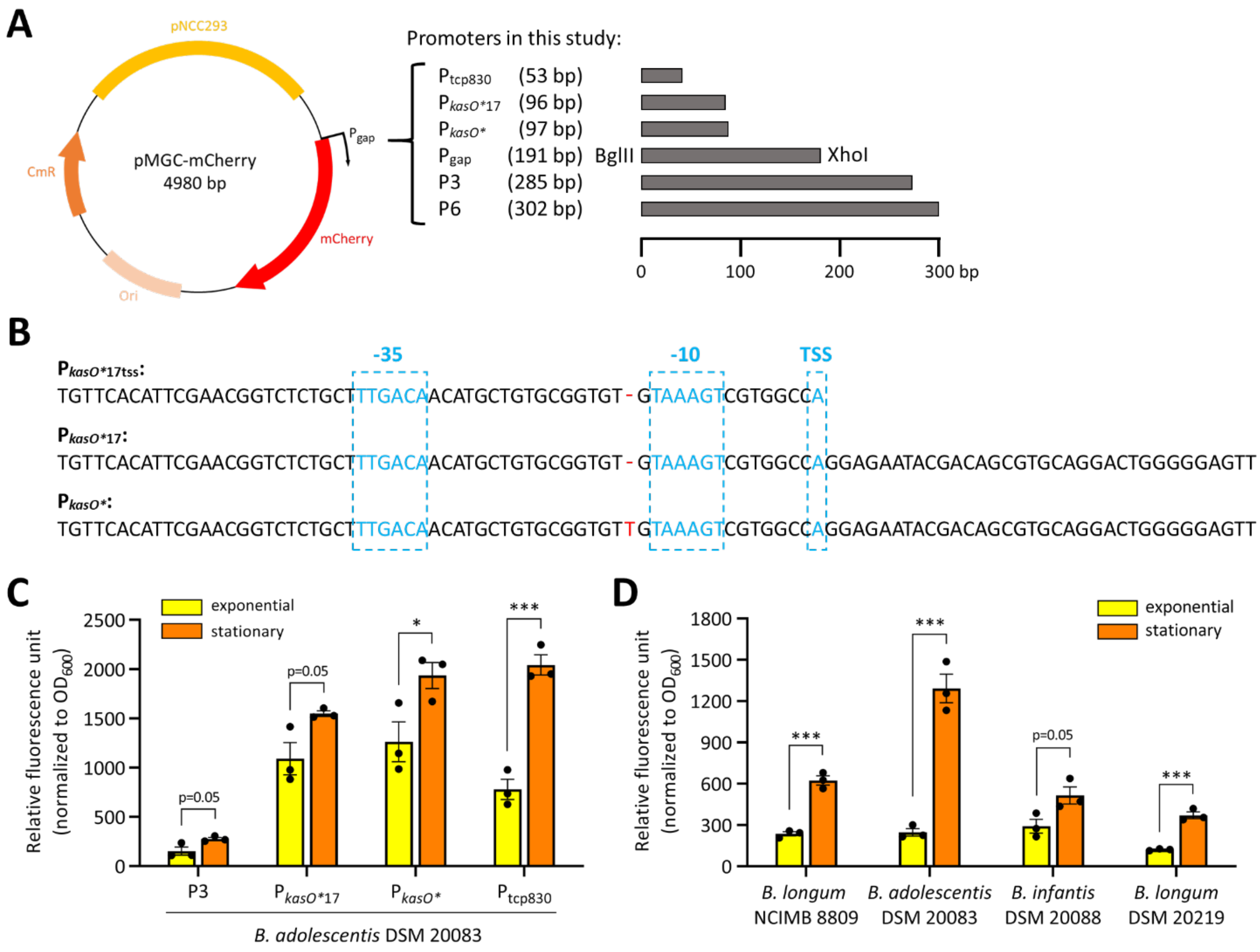

Figure S2. Detailed information for derived mCherry-expressing plasmids and relative promoters strength across *Bifidobacterium* sp.. (A) Genetic map of pMGC-mCherry plasmid with different promoters used in this study. (B) Sequence alignment of P_{kasO*} and its variants, $P_{kasO*17}$ and $P_{kasO*17tss}$. The blue dashed boxes indicated the -10, -35 regions and transcription start site (TSS). $P_{kasO*17tss}$ is truncated at the TSS site for sgRNA expression. (C) Relative expression levels of indicated promoters in *B. adolescentis* DSM 20083 in exponential and stationary phase cultures. (D) Relative mCherry fluorescence levels in exponential and stationary phase cultures of *Bifidobacterium* sp. transformed with pMGC-mCherry. (n=3; error bars, s.e.m.; *P<0.05, ***P<0.001)

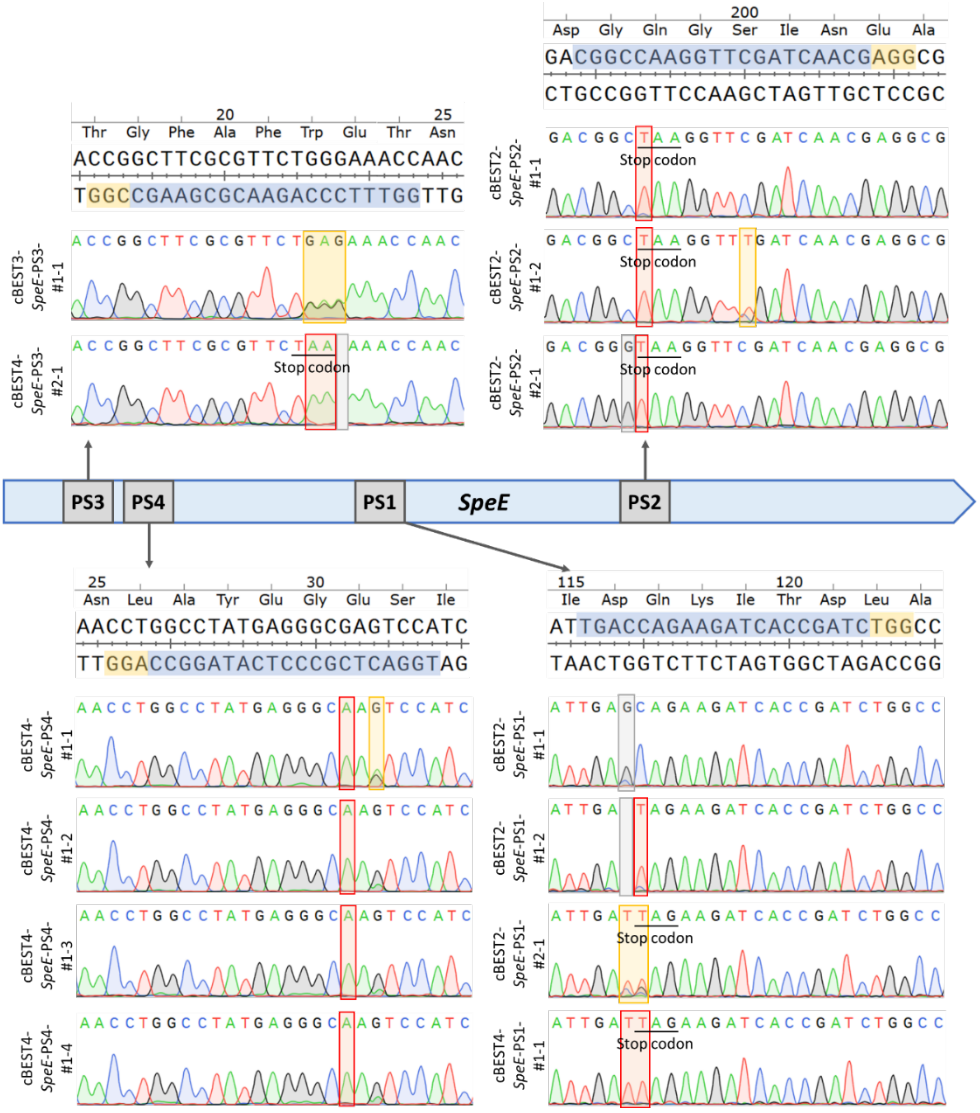

Figure S3. Successful gene editing within four protospacers of the *SpeE* gene in *B. longum* NCIMB 8809. Sequencing results confirmed precise introduction of stop codon due to successful C-to-T edits (red frames) in indicated protospacers. Orange frames represent mixed colonies consisting of wildtype and edited cells. Grey frames represent undesired point mutation or deletion. The top row displays the wild type reference sequences. Protospacers *SpeE*-PS1 and *SpeE*-PS2 targeted the forward strand, while *SpeE*-PS3 and *SpeE*-PS4 targeted the reverse strand.

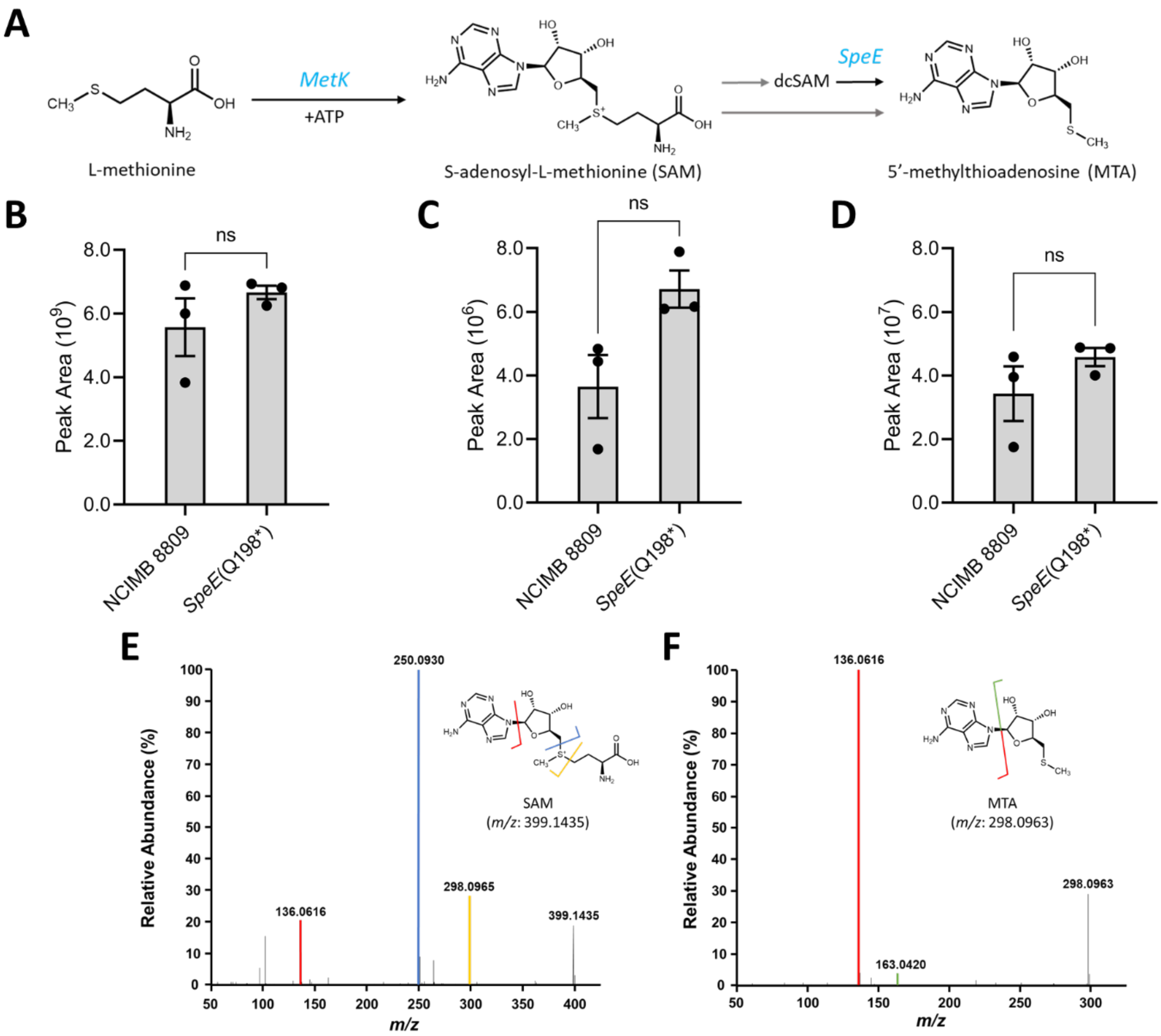

Figure S4. Evaluation of MTA regulation in the *SpeE*(Q198*) strain. (A) Biosynthetic pathway of MTA with the indicated genes in *B. longum* NCIMB 8809. LC-MS/MS analysis depicting peak areas for (B) L-methionine, (C) S-adenosyl-L-methionine (SAM) and (D) MTA in both wild-type and the *SpeE*(Q198*) strain. dcSAM was not detected. (n=3, biological triplicates; error bars, s.e.m.; ns, not statistically significant) (E and F) Annotation of MS/MS fragmentation patterns for SAM and MTA.

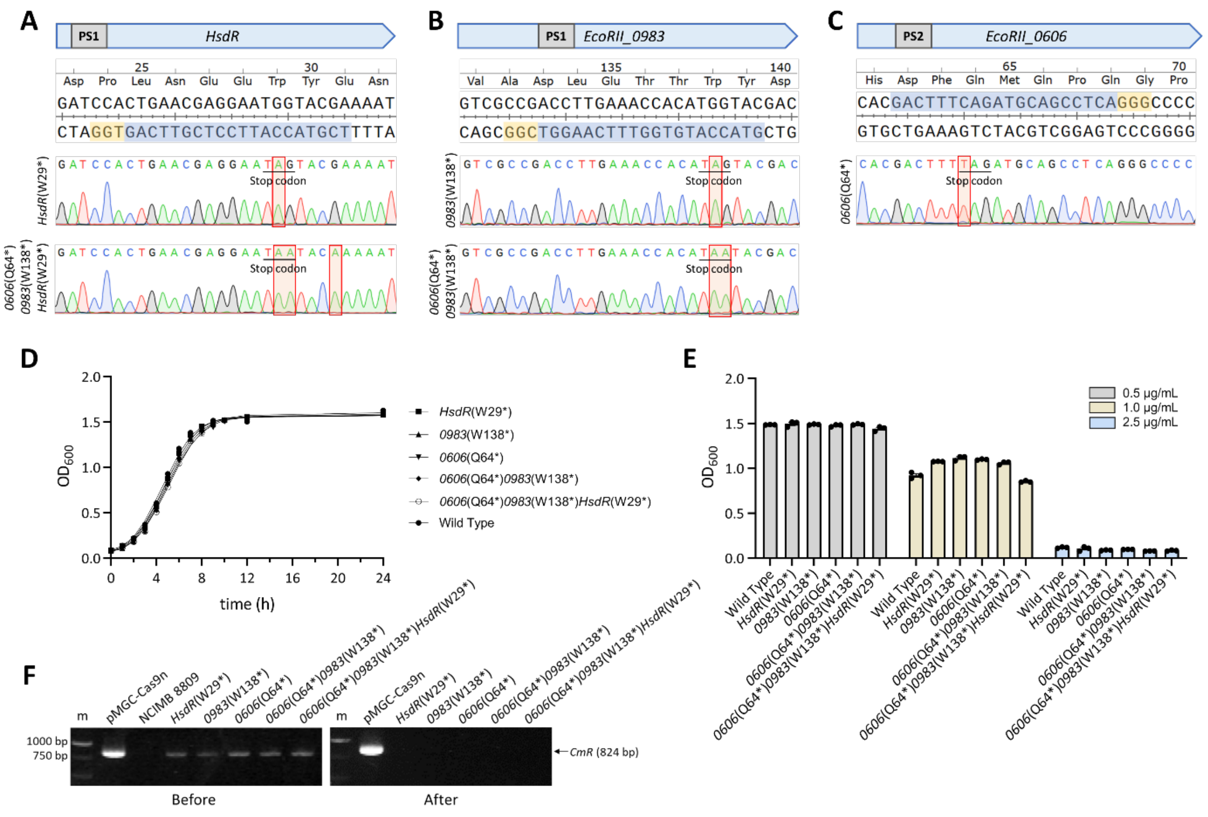

Figure S5. Obtaining five REase-mutant strains derived from *B. longum* NCIMB 8809 with their growth curves and chloramphenicol sensitivity test. (A to C) Sequencing results confirmed precise introduction of stop codon due to C-to-T mutations (red frames). The top row displays the wild type sequences for reference and the mutant strains are labeled. (D) Bacterial growth in MRSC broth over 24 h. (n=3; error bars, s.e.m.) (E) Antibiotics sensitivity test conducted at the indicated chloramphenicol concentrations for 24 h. (n=3; error bars, s.e.m.) (F) Colony PCR to monitor presence or loss of editing plasmids before and after plasmid curing, respectively. Primers were designed to target the <u>*CmR*</u> chloramphenicol resistant gene.

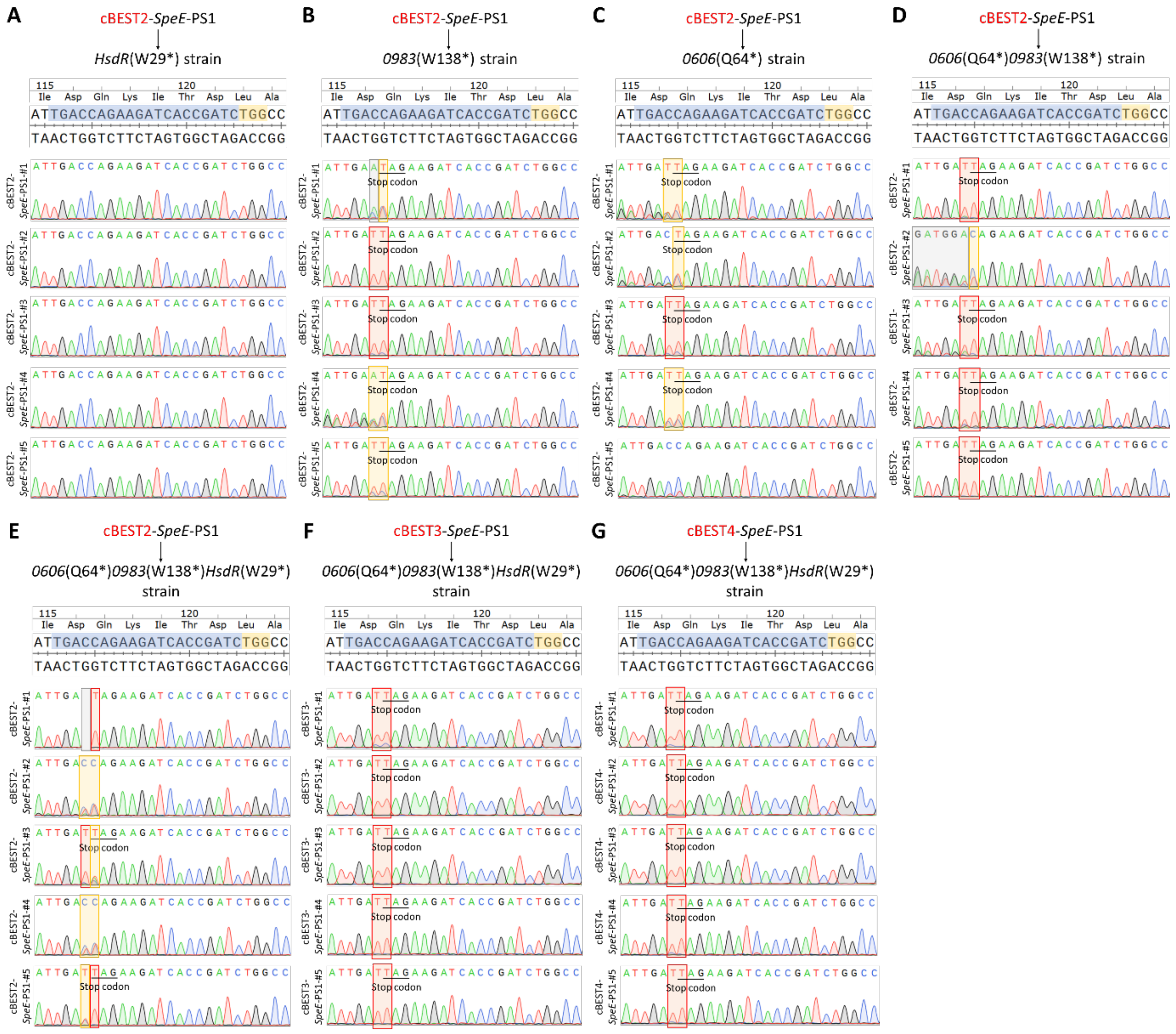

Figure S6. Evaluation of gene editing patterns for REase mutant strains derived from *B. longum* NCIMB 8809 using various cBEST-*SpeE*-PS1 plasmids. Sequencing results confirmed precise introduction of stop codon due to successful C-to-T conversions (red frames) in the *SpeE*-PS1 protospacer. Orange frames represent mixed colonies consisting of wildtype and edited cells. Grey frames represent undesired point mutation or deletion. The top row displays the wild type sequences for reference. (A to E) Sequencing details for transforming cBEST2-*SpeE*-PS1 plasmid across REase mutant strains. (E to G) Sequencing details for transforming three cBEST-*SpeE*-PS1 plasmids into the *0606*(Q64*)*0983*(W138*)*HsdR*(W29*) strain.

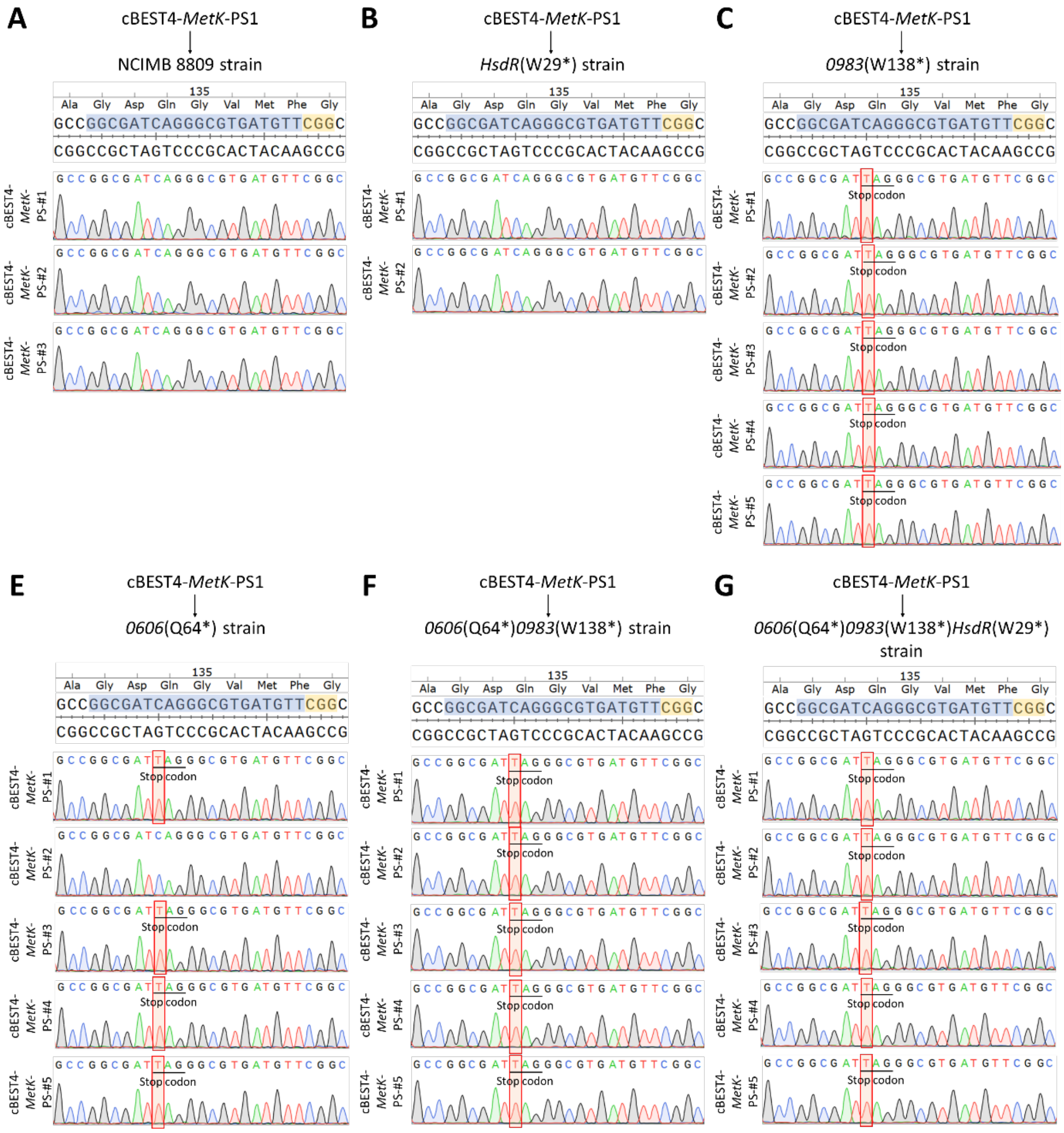

Figure S7. Evaluation of gene editing patterns for REase mutant strains derived from *B. longum* NCIMB 8809 using cBEST4-*MetK*-PS1 plasmid. Sequencing results confirmed precise introduction of stop codon due to successful C-to-T conversions (red frames) for the indicated strains. The top row displays the wild type sequences for reference.

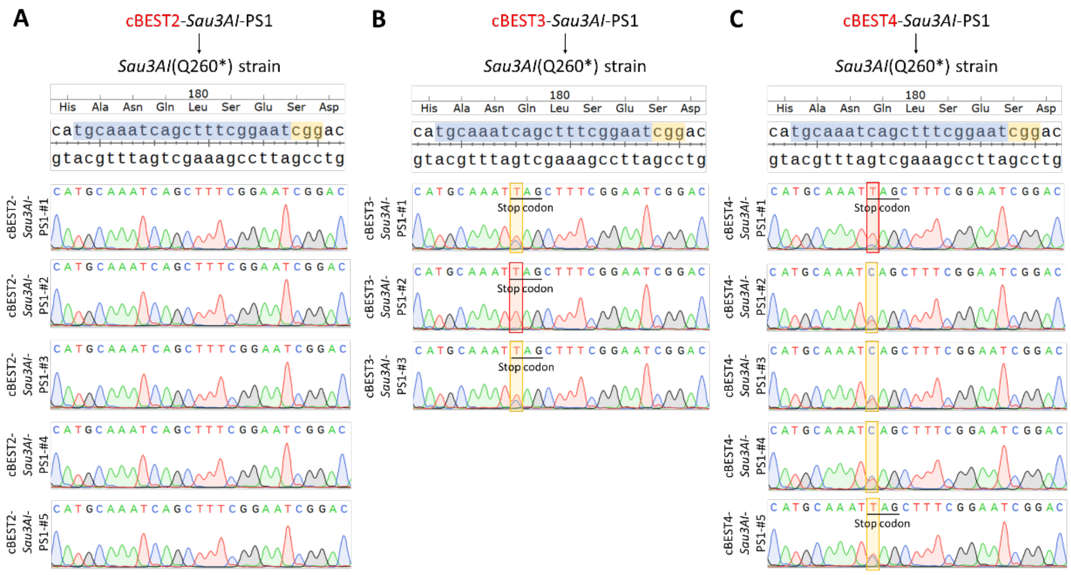

Figure S8. Gene editing patterns using various cBEST-*Sau3AI*-PS1 plasmids in the *Sau3AI*(Q260*) strain. (A-C) Sequencing results confirmed precise introduction of stop codon (black line) due to successful C-to-T conversions (red frame) in the *Sau3AI*-PS1 protospacer. The orange frames represent mixed edited sites. The top row displays the wild type sequences for reference. For (B), only three transformants were obtained on the agar plate for sequencing.

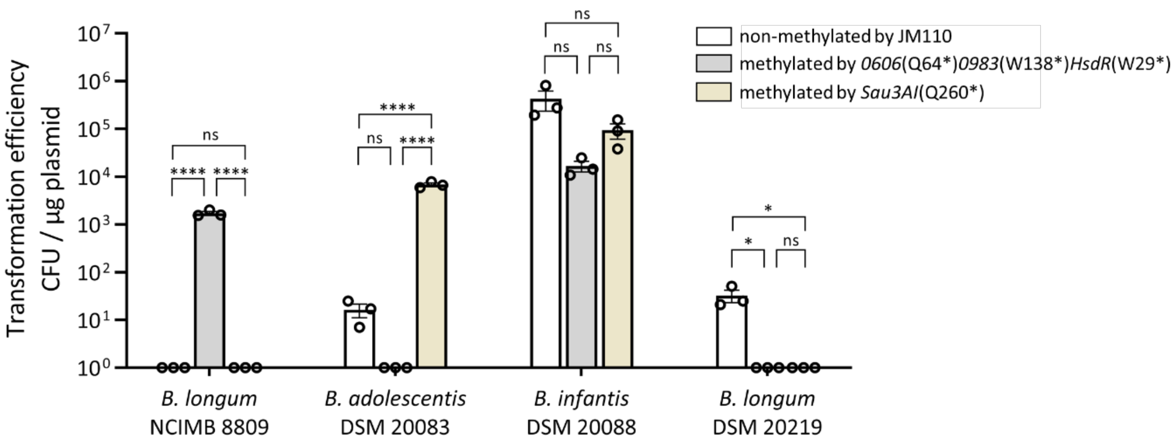

Figure S9. Comparison of transformation efficiencies using non-methylated or methylated pMGC-Cas9n plasmids across *Bifidobacterium* spp.. The methylated pMGC-Cas9n were derived from the *0606*(Q64*)*0983*(W138*)*HsdR*(W29*) strain or the *Sau3AI*(Q260*) strain followed the same experimental workflow in Figure 4A. (n=3, biological triplicates; error bars, s.e.m.; ns, not significant, *P<0.05, ****P<0.0001)

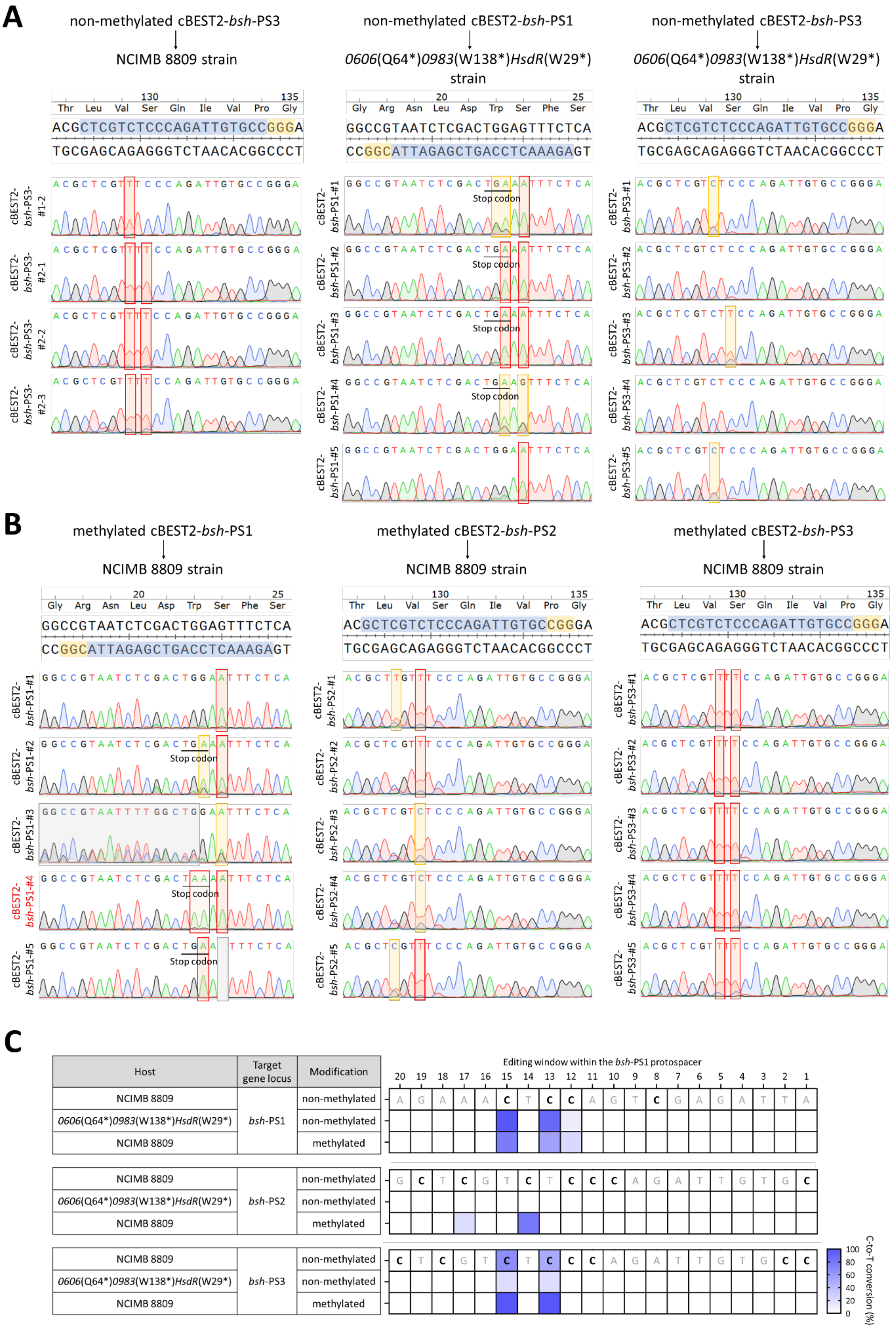
A
non-methylated cBEST2-bsh-PS3
NCIMB 8809 strain
non-methylated cBEST2-bsh-PS1
0606(Q64*)0983(W138*)HsdR(W29*) strain
non-methylated cBEST2-bsh-PS3
0606(Q64*)0983(W138*)HsdR(W29*) strain
Stop codon
B
methylated cBEST2-bsh-PS1
NCIMB 8809 strain
methylated cBEST2-bsh-PS2
NCIMB 8809 strain
methylated cBEST2-bsh-PS3
NCIMB 8809 strain
Stop codon
C
Host
Target gene locus
Modification
Editing window within the bsh-PS1 protospacer
NCIMB 8809
0606(Q64*)0983(W138*)HsdR(W29*)
bsh-PS1
bsh-PS2
bsh-PS3
non-methylated
methylated
C-to-T conversion (%)

Figure S10. Gene editing patterns using various non-methylated/methylated cBEST2-*bsh* plasmids in wild type and *0606*(Q64*)*0983*(W138*)*HsdR*(W29*) *B. longum* NCIMB 8809 strain. (A) Gene editing patterns for non-methylated cBEST2-*bsh* plasmids into wild type or *0606*(Q64*)*0983*(W138*)*HsdR*(W29*) *B. longum* NCIMB 8809 strains. (B) Gene editing patterns for methylated cBEST2-*bsh* plasmids into indicated NCIMB 8809 strains. Sequencing results confirmed introduction of stop codon (black line) due to successful C-to-T edits (red frame) in three indicated protospacers. The orange frames represent mixed edited sites and the grey frames represent undesired point mutation or deletion. (C) Editing windows for indicated conditions.

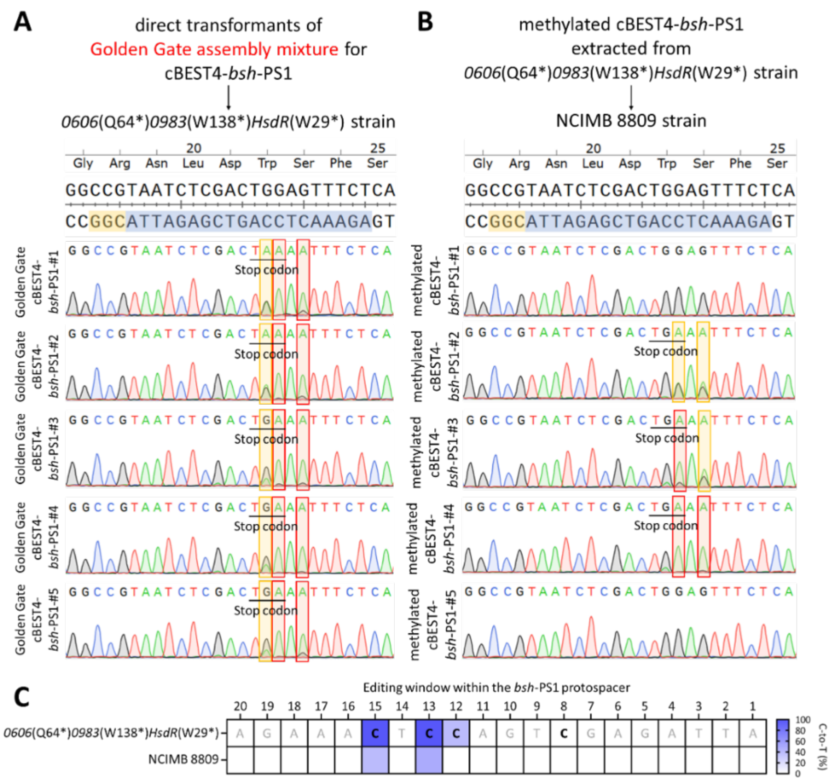

Figure S11. Gene editing patterns using the two methods in Figure 4C. Sequencing results confirmed introduction of stop codon (black line) due to successful C-to-T edits (red frame) in three indicated protospacers. The orange frames represent mixed edited colony, where the colony contained both wild type and edited cells. (A) Gene editing patterns of Golden Gate assembly mixture for constructing cBEST4-*bsh*-PS1 plasmid through the *0606*(Q64*)*0983*(W138*)*HsdR*(W29*) strain. (B) Gene editing patterns for methylated cBEST4-*bsh*-PS1 plasmid into *B. longum* NCIMB 8809. (C) Editing window for the two strains.

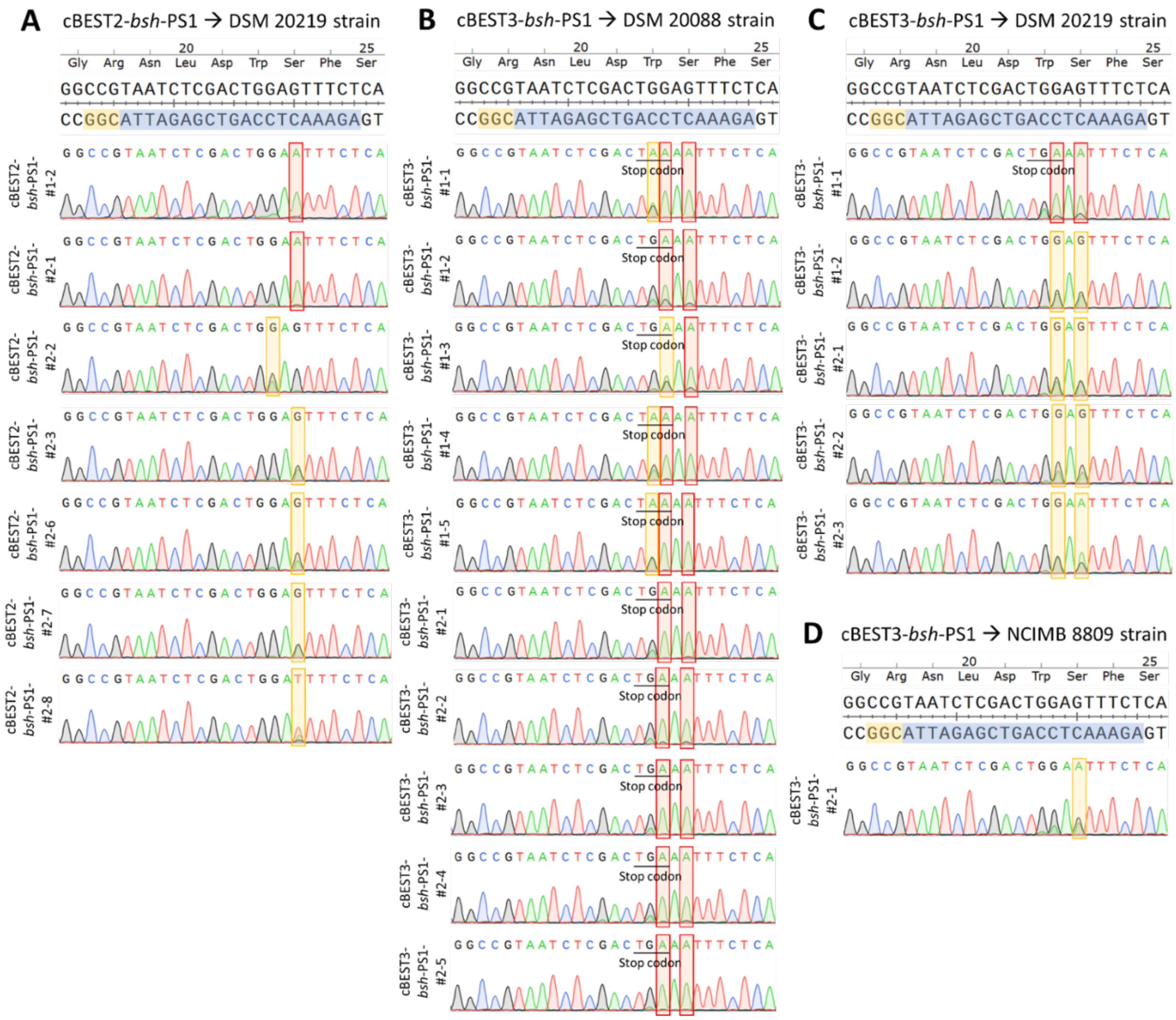

Continued on next page.

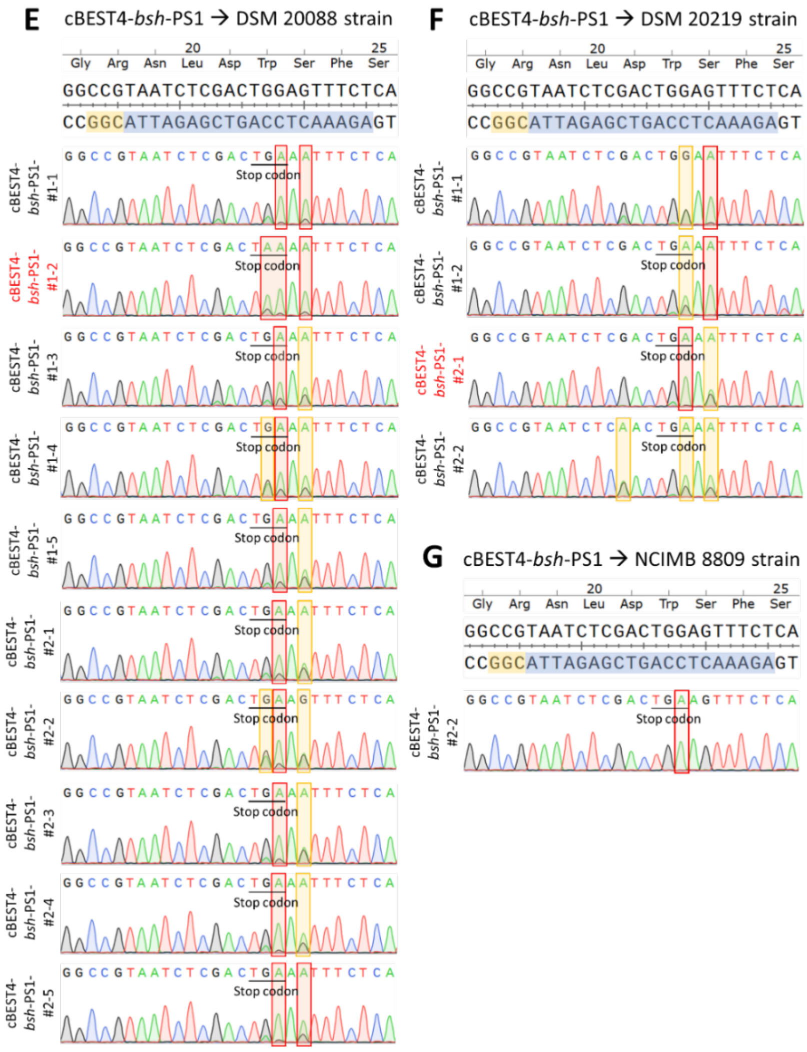

Figure S12. Gene editing patterns for various *Bifidobacterium* strains using three cBEST-*bsh*-PS1 plasmids in Figure 5B. Sequencing results confirmed precise introduction of stop codon (black line) due to successful C to T conversions (red frame). The orange frames represent mixed edited sites. Gene editing patterns for (A) cBEST2-*bsh*-PS1 plasmid transforming into DSM 20219, (B to D) cBEST3-*bsh*-PS1 plasmid transforming into three strains and (E to G) cBEST4-*bsh*-PS1 plasmid transforming into three strains. Strains using scarlet letters were used for functional studies after plasmid curing.

A

compound	RT (min)	Q1 (Da)	Q3 (Da)	time (ms)	DP (V)	EP (V)	CE (V)	CXP (V)
TCA	3.4	533	462	50	62	10	33	18
			516				17	22
			498				19	21
CA	4.18	426	355	50	75	9	29	14
			373				21	15
			91				98	10
CA-d4	4.16	430	359	50	87	9	28	14
			377				21	15
DCA	5.53	410	357	50	68	11	18	14
			375				15	15
			339				23	15

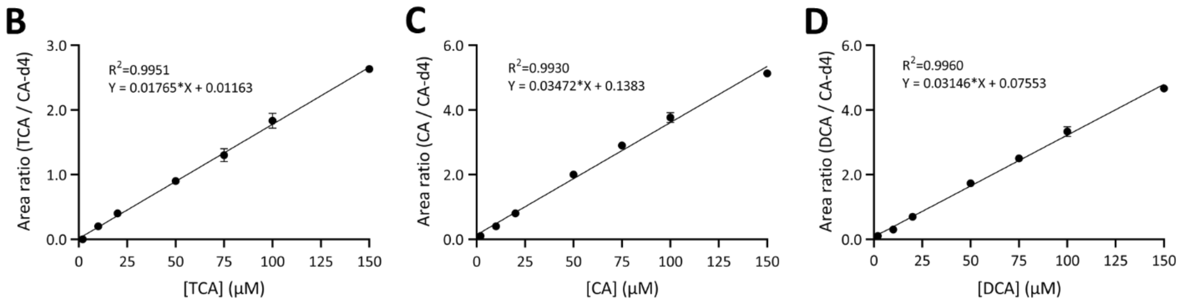

Figure S13. QqQ multiple reaction monitoring (MRM) method and the calibration curves. (A) MRM tuning parameters containing parent ion (Q1), daughter ion (Q2), ion dwell time, declustering potential (DP), entrance potential (EP), collision energy (CE) and cell exit potential (CXP). RT, retention time. Calibration curves as well as their equations and R^2 values for (B) TCA, (C) CA and (D) DCA. Area ratios were defined by the peak area normalized to peak area of CA-d4 internal standard.

compound	Q1 (Da)	Q3 (Da)	time (ms)	DP (V)	EP (V)	CE (V)	CXP (V)
MET	150	56<br>104<br>133	50	50	14	21<br>12<br>13	7<br>7<br>9
MET-IS (13C,d3)	154	108<br>55<br>137	50	61	15	16<br>22<br>14	9<br>7<br>10
SAM	399	250<br>298<br>136	50	70	10	19<br>17<br>33	9<br>12<br>9
MTA-IS (d3)	301	136<br>119<br>64	50	50	6	25<br>67<br>51	11<br>9<br>8

Figure S14. QqQ MRM tuning parameters for methionine-derived metabolites. MRM tuning parameters containing parent ion (Q1), daughter ion (Q2), ion dwell time, declustering potential (DP), entrance potential (EP), collision energy (CE) and cell exit potential (CXP).

Supplementary Methods

Metabolomics for *Bifidobacterium* strains (Figure S4)

For the analysis of methionine-related metabolic pathways in Figure S4, 100 µL of overnight cultures were plated on MRSC agar plate. After 18 h incubation, bacteria were scraped and extracted with 1 mL 80% LC-grade methanol. Samples were homogenized by ultrasonication (25 W, 20 kHz, 1 min) and then centrifuged for 13000 rpm 15 min at 4 °C. Supernatants were transferred to autosampler vials for untargeted LC-MS/MS analysis.

LC-MS/MS was performed on a Dionex U3000 UPLC system coupled with a Thermo Scientific Q Exactive Plus equipped with heated electrospray ionization (HESI). 3 µL of sample was injected and separated using Acquity HSS T3 column (2.1×100 mm, 1.7 µm) at 40 °C. Mobile phase A was 0.1% formic acid in deionized water while mobile phase B was 0.1% formic acid in acetonitrile. The elution separation gradient was as follows: 5% B for 1 min, linear increase to 95% B at 8 min, held at 95% B for 3 min, decreased linearly to 5% B at 12 min and held for another 3 min. Mass spectrometer parameters were also as follows: ionization voltage 3.5 kV (positive mode), capillary temperature 250 °C and sheath gas flow rate of 25 µL/min. The scan method was operated in Top10 data-dependent acquisition (ddMS2) with a normalized collision energy of 30.

Antibiotic susceptibility tests

Overnight seed cultures were diluted 100-fold in MRSC broth supplemented with various chloramphenicol concentrations in a 96-well plate and incubated anaerobically at 37 °C for 24 h. Cell density was measured by optical density at 600 nm (OD_{600}) using a Synergy H1 Microplate Reader from BioTek.
